# A thiopyrylium salt for PET/NIR‐II tumor imaging and image‐guided surgery

**DOI:** 10.1002/1878-0261.12674

**Published:** 2020-04-07

**Authors:** Xiao Zhang, Bingbing Ding, Chunrong Qu, Huiling Li, Yu Sun, Yongkang Gai, Hao Chen, Hanyi Fang, Kun Qian, Yongxue Zhang, Zhen Cheng, Xiaoli Lan

**Affiliations:** ^1^ Department of Nuclear Medicine Union Hospital Tongji Medical College Huazhong University of Science and Technology Wuhan China; ^2^ Molecular Imaging Program at Stanford Bio‐X Program, and Department of Radiology Canary Center at Stanford for Cancer Early Detection Stanford University CA USA; ^3^ Hubei Key Laboratory of Molecular Imaging Wuhan China

**Keywords:** image‐guided surgery, multimodal imaging, positron‐emission tomography, second near‐infrared window

## Abstract

All tumor imaging modalities have resolution limits below which deeply situated small metastatic foci may not be identified. Moreover, incomplete lesion excision will affect the outcomes of the patients. Scintigraphy is adept in locating lesions, and second near‐infrared window (NIR‐II) imaging may allow precise real‐time tumor delineation. To achieve complete excision of all lesions, multimodality imaging is a promising method for tumor identification and management. Here, a NIR‐II thiopyrylium salt, XB1034, was first synthesized and bound to cetuximab and trans‐cyclooctene (TCO) to produce XB1034‐cetuximab‐TCO. This probe provides excellent sensitivity and high temporal resolution NIR‐II imaging in mice bearing tumors developed from human breast cancer cells MDA‐MB‐231. To enable PET imaging, ^68^Ga‐NETA‐tetrazine is subsequently injected into the mice to undergo a bio‐orthogonal reaction with the preinjected XB1034‐cetuximab‐TCO. PET images achieved in the tumor models using the pretargeting strategy are of much higher quality than those obtained using the direct radiolabeling method. Moreover, real‐time NIR‐II imaging allows accurate tumor excision and sentinel lymph node mapping. In conclusion, XB1034 is a promising molecular imaging probe for tumor diagnosis and treatment.

## Graphical abstract

Here, a NIR‐II thiopyrylium salt, XB1034, was first synthesized and bound to cetuximab and trans‐cyclooctene (TCO) to produce XB1034‐cetuximab‐TCO. XB1034‐cetuximab‐TCO manifested promising NIR‐II imaging activity, and the follow‐up injected Tz‐NETA‐^68^Ga probe showed encouraging efficiency for tumor quantification. This targeted dual‐modality probe demonstrated the feasibility of clinical image‐guided operation in real time.

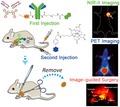

**Abbreviations**
DCE: dichloroethane
EGFR: epidermal growth factor receptor
H&E: hematoxylin and eosin
HOMOs: highest occupied molecular orbitals
HPLC: high‐performance liquid chromatography
ICG: indocyanine green
LP: long pass
LUMOs: lowest unoccupied molecular orbitals
MALDI‐TOF MS: matrix‐assisted laser desorption/ionization time‐of‐flight mass spectrometry
MTT: methyl thiazolyl tetrazolium
NETA‐Tz: tetrazine‐(2,2′‐((6‐amino‐1‐(4,7‐bis(carboxymethyl)‐1,4,7‐triazonan‐1‐yl)hexan‐2‐yl)azanediyl)diacetic acid
NIR‐II: second near‐infrared window
NMR: nuclear magnetic resonance
PET: positron‐emission tomography
PL: photoluminescence
QY: fluorescence quantum yield
*S*_0_: ground state
*S*_1_: first excited state
SLN: sentinel lymph node
SNR: signal‐to‐noise ratio
TCO: trans‐cyclooctene
Tz: tetrazine

## 1 Introduction

Molecular imaging provides a noninvasive method for effectively monitoring biomolecules at the cellular or subcellular level (Tahara *et al.*, 2014). Each modality, including radiopharmaceutical, magnetic resonance, targeted ultrasound, and optical imaging, has its advantages and disadvantages. Multimodal imaging integrates the advantages of various modalities and minimizes their shortcomings (Sheng *et al.*, 2018; Weissman *et al.*, 2013). PET imaging is outstanding in locating lesions and detecting metastasis with nearly unlimited tissue penetration depth and excellent sensitivity (Lee *et al.*, 2014), but PET is not practical for real‐time and continuous observation (Hernandez *et al.*, 2016). Fluorescence imaging has attracted increasing attention due to its high temporal resolution, spatial resolution, and real‐time tumor delineation (Li *et al.*, 2014; Zhang *et al.*, 2020). It is therefore an ideal modality to pair with PET.

However, most traditional optical imaging methods have some shortcomings in *in vivo* studies. Autofluorescence caused by the fur and normal tissues of experimental animals and background noise could result in a low signal‐to‐noise ratio (SNR) (Hong *et al.*, 2012). The penetration depth of most fluorescence wavelengths is also limited (Shou *et al.*, 2018). To overcome these limitations, probes with emissions in the second near‐infrared window (NIR‐II, 1000–1700 nm) for multimodal imaging are being developed (Cheng *et al.*, 2017; He *et al.*, 2018; Shou *et al.*, 2017) and gaining increasing attention in the application of optical imaging (Feng *et al.*, 2017; Li *et al.*, 2018b; Wan *et al.*, 2018; Yang *et al.*, 2018a, 2018b,2018a, 2018b; Zhu *et al.*, 2018a). The penetration depth of NIR‐II fluorescence could reach up to 10 mm (Benhao *et al.*, 2018; Shao *et al.*, 2016; Wang *et al.*, 2019). And organic dyes have low toxicity with good biocompatibility and pharmacokinetics, which makes them appealing imaging agents in clinical applications (Antaris *et al.*, 2016, 2017; Lei *et al.*, 2019; Zhang *et al.*, 2016). The polymethine thiopyrylium salt derivates are becoming one of the most important NIR‐II small‐molecule dyes for their high molar absorption coefficient and both long absorption and emission wavelength up to 1300 nm (Li *et al.*, 2018a; Wang *et al.*, 2019). While applied in biological imaging, these dyes are tended to form into nanoparticles or used directly without specific‐targeted modification (Cosco *et al.*, 2017; Tao *et al.*, 2013; Xie *et al.*, 2019; Yufu *et al.*, 2018). Our previously reported polymethine thiopyrylium salt 5H5 has achieved excellent specific‐targeted tumor imaging after conjugated with an RGD‐based peptide (Ding *et al.*, 2019). Unfortunately, the pursuit of long emission wavelength sacrifices the fluorescence quantum yield (QY) and chemical stability. Herein, a new small‐molecule organic dye, namely XB1034, was achieved with improved molar absorption coefficient and QY after shortening the polymethine chain. Surprisingly, as the polymethine chain shortened, XB1034 can afford more modification strategies, such as click chemistry and amide condensation reactions, attributing to the improvement of chemical stabilities. To perform high‐efficiency targeted biological imaging, XB1034 was introduced into monoclonal antibody cetuximab (~ 150 kDa) to construct a probe XB1034‐cetuximab targeting epidermal growth factor receptor (EGFR).

**Scheme 1:**
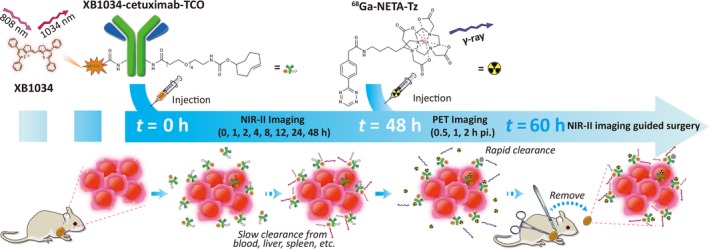
The schematics of multimodal PET and NIR‐II imaging.

To achieve NIR‐II/PET dual‐modal imaging, the fluorescence dye and radiotracer are usually combined into an integrated whole (Sun *et al.*, 2018; Zhang *et al.*, 2019). However, they are limited to get ideal radionuclide imaging for the nuclides with short half‐life time (Zhang *et al.*, 2018). Recently, a pretargeting technique using bio‐orthogonal reactions was reported to afford higher image quality and reduced nontargeted radiation dosed to patients and surgeons (Lutje *et al.*, 2014; van Duijnhoven *et al.*, 2015). Specifically, the strategy uses an inverse‐electron‐demand Diels–Alder reaction between trans‐cyclooctene (TCO) and tetrazine (Tz) (Meyer *et al.*, 2018; Zeglis *et al.*, 2013). Inspired by that, our strategy is to modify XB1034‐cetuximab with TCO to afford XB1034‐cetuximab‐TCO and get the NIR‐II tumor imaging (Adumeau *et al.*, 2016; Zeglis *et al.*, 2015). The subsequent injection of radiolabeled Tz‐(2,2′‐((6‐amino‐1‐(4,7‐bis(carboxymethyl)‐1,4,7‐triazonan‐1‐yl)hexan‐2‐yl)azanediyl)diacetic acid (L‐NETA, a metal chelating agent, Ludwig *et al.*, 2017) enables click conjugation of TCO and Tz, accompanied by rapid renal clearance of unbound ^68^Ga‐NETA‐Tz. After ten half‐lives of radioisotope (half‐life of ^68^Ga: 68 min), NIR‐II imaging‐guided surgery would be performed (Scheme 1).

## 2 Materials and methods

### 2.1 Synthesis of XB1034

The preparations of probes are listed in Scheme S1. The synthesis of thiopyrylium **a** is described before (Ding *et al.*, 2019). Thiopyrylium **a** (0.2 mmol, 86.0 mg), N,N'‐Diphenylformamidine **b** (0.1 mmol, 19.62 mg), and anhydrous sodium acetate (0.1 mmol, 8.0 mg) were stirred in acetic anhydride (10 mL) for 120 min at 70 °C. The mixture was washed with ethyl ether and then desiccated. Finally, XB1034 (58 mg) was obtained after purified by high‐performance liquid chromatography (HPLC) eluted with acetonitrile/water (containing 0.1% TFA). Yield: 74%; ^1^H NMR (400 MHz, Acetonitrile‐*d*3) δ 7.70 (d, *J* = 8.8 Hz, 4H), 7.52 (s, 1H), 7.50 (10H), 7.41 (s, 2H), 7.06 (d, *J* = 8.9 Hz, 4H), 4.78 (d, *J* = 2.3 Hz, 4H), 3.02 (8H), 2.88 (t, *J* = 2.3 Hz, 2H); ^13^C NMR (101 MHz, CD_3_CN) δ 161.6, 161.0, 147.5, 146.9, 146.7, 139.2, 138.0, 130.7, 129.9, 129.2, 129.1, 129.1, 128.9, 127.1, 116.7, 79.0, 77.5, 56.9, 31.6, 30.7; MALDI‐TOF mass: *m/z* calcd for C_47_H_35_O_2_S_2_^+^, 695.21 [M‐BF_4_]^+^; found: 695.11.

The theoretical characteristics of XB1034 were evaluated by commercial software (gaussian 09^®^; Gaussian Inc., Wallingford, CT, USA). The optimized geometries of ground state (*S*_0_) and first excited state (*S*_1_), and highest and lowest unoccupied molecular orbitals (HOMOs and LUMOs) were acquired for predicting optical properties using time‐dependent density functional theory calculations. The fluorescence emission wavelength of XB1034 was obtained using the TD OPT B3LYP/6‐31G(d) cpcm = solvent = acetonitrile method.

### 2.2 Synthesis of XB1034 NHS ester, XB1034‐cetuximab, and XB1034‐cetuximab‐TCO

XB1034 (3.9 mg, 5 μmol) was added to a solution of azido‐PEG_8_‐NHS (3.4 mg, 6 μmol) in DMF (0.5 mL), followed by stirring with copper sulfate (0.08 mg, 0.5 μmol) and sodium ascorbate (0.1 mg, 0.5 μmol) for 2 h. And diethyl ether was subsequently added to the mixture. Finally, XB1034‐NHS was collected and purified by HPLC. MALDI‐TOF mass *m*/*z*: [M]^+^ calcd for C_93_H_115_N_8_O_26_S_2_^+^, 1823.74; found, 1823.60. To test the photostability of XB1034‐NHS, the sample was exposed to continuous 808‐nm laser irradiation for 2 h and the fluorescence intensity was measured.

XB1034 NHS ester (200 µg in 20 µL DMSO) was added to cetuximab solution (870 µg, purified by Zeba column) and stirred for 3 h. We used 3K MWCO filter to purify XB1034‐cetuximab and further identify with HPLC. XB1034‐cetuximab‐trans‐TCO was synthesized by stirring XB1034‐cetuximab and TCO‐PEG_4_‐NHS ester (60 µg) for 3 h (purified by 3K MWCO filter).

### 2.3 Preparation of ^68^Ga‐NETA‐Tz and ^68^Ga‐NETA‐XB1034‐cetuximab

We used 0.05 m HCl to elute ^68^Ga (222 MBq, 500 µL), and sodium acetate buffer (1.25 m, 150 µL) was added to regulate pH to 4.0. Followed by the addition of 6 nmol NETA‐Tz, the mixture was stirred at 90 °C for 12 min to obtain ^68^Ga‐NETA‐Tz. For the direct imaging, XB1034‐cetuximab‐trans‐TCO was mixed with ^68^Ga‐NETA‐Tz for 20 min to prepare ^68^Ga‐NETA‐cetuximab‐XB1034 (purified by PD‐10 gel column).

### 2.4 Cell culture and identification

Human breast cancer cell lines MDA‐MB‐231 and MCF‐7, and NIH 3T3 fibroblasts were maintained in DMEM containing 10% FBS at 37 °C. The expression of EGFR in MDA‐MB‐231 and MCF‐7 cells was identified by western blot. Cell uptake assay was measured to test the binding affinity of XB1034‐cetuximab‐TCO. The experiment was carried out in 4 × 10^6^ cells and then incubated with serum‐free DMEM containing XB1034‐cetuximab‐TCO (50 nm) with/without excessive cetuximab (2.5 μm) for 4 h, respectively. The cells were washed three times with PBS and further digested with pancreatic enzyme to get fluorescence intensities of digestions. A quantity of 1 × 10^4^ 3T3 fibroblasts per well were planted into 96‐well plates, followed by various concentration (1, 2, 5, 10, 25, 50, and 100 µg·mL^−1^, *n* = 5 each concentration) of XB1034‐NHS incubation overnight. And the samples were treated with 1 mm methyl thiazolyl tetrazolium (MTT) for an additional 4 h. The formazan crystals were dissolved in 150 µL DMSO per well, and the absorbance of the samples was tested at 490 nm to calculate cell viability.

### 2.5 Animal models

The animal studies were approved by the Animal Care Committee of Tongji Medical College, Huazhong University of Science and Technology. The mice were purchased from Beijing HFK Bioscience Co. Ltd (Beijing, China). A total of 27 nude mice (4–6 weeks) were received a subcutaneous injection of MDA‐MB‐231 cells (21 mice) or MCF‐7 (3 mice) in the shoulder. The mice were used for *in vivo* studies after injections for three weeks.

### 2.6 *In vivo* NIR‐II imaging

XB1034‐cetuximab‐TCO (each 150 µg, 150 µL) were injected into the mice bearing MDA‐MB‐231 (*n* = 12) or MCF‐7 (*n* = 3) tumors via the tail vein under 2.5% isoflurane in oxygen for anesthetization. NIR‐II imaging was performed in a home‐built NIR‐II imaging system using a 1000‐nm long‐pass (LP) filter under 808‐nm excitation. Blood pool imaging was immediately acquired when the probe was injected into the mice. The vessel diameters were calculated from the corresponding full width at half maximum of the peaks. Blocking study was performed with unlabeled cetuximab (1.5 mg in 200 μL PBS) coinjection (*n* = 3). The whole‐body imaging in the NIR‐II window was acquired at different time points: 0.5, 1, 2, 4, 8, 12, and 48 h postinjection. The excitation intensity of the irradiation was kept below 100 mW·cm^−2^.

### 2.7 Small‐animal PET scanner imaging and biodistribution studies

Forty‐eight hours after XB1034‐cetuximab‐TCO injection, the mice were injected intravenously with ^68^Ga‐NETA‐Tz (14.8–22.2 MBq, 150 µL), and PET images were acquired under 2.5% isoflurane in oxygen for anesthetization. The biodistribution studies were carried out at 0.5, 1, and 2 h after ^68^Ga‐NETA‐Tz injection in MDA‐MB‐231 (*n* = 3 per group) and 2 h in MCF‐7 tumor‐bearing mice (*n* = 3). The interested tissues were collected, weighed, and measured with an automated γ counter to calculate the percentage of injected dose per gram of tissue (%ID·g^−1^). ^68^Ga‐NETA‐cetuximab‐XB1034 (14.8–22.2 MBq, 150 μL) was also injected into MDA‐MB‐231 models to get the direct EGFR‐specific PET images (*n* = 3).

### 2.8 NIR‐II image‐guided surgery of tumors

We used NIR‐II to guide surgical resection of the tumor tissue. The mice were anesthetized at 60 h postinjection of antibody‐modified compounds and nearly 10 h after PET imaging. Tumor resection was performed under NIR‐II image guidance. Tumor‐to‐background ratios (*n* = 3) were calculated using imagej software (Bethesda, MD, USA) before and after removal. The removed tumors and surrounding tissues were stained with hematoxylin and eosin (H&E).

### 2.9 NIR‐II imaging‐guided lymph node mapping

Before imaging, the mice (*n* = 3) were anesthetized with 2.5% isoflurane inhalation. A total of 50 µL XB1034‐cetuximab‐TCO (30 µg) was injected into the right foot pad to visualize the lymphatic drainage and lymph nodes. To pattern the standard sentinel lymph node (SLN) biopsy process of clinical use, nude mice with MDA‐MB‐231 tumors (*n* = 3) were applied. To trace the lymphatic pathway in real time, XB1034‐cetuximab‐TCO (30 µg) was injected intradermally near the tumor. At 10 min postinjection, the SLNs were excised with the aid of NIR‐II imaging and identified by H&E staining.

### 2.10 Statistical analysis

Quantitative data were expressed as mean ± standard deviation (SD), and differences between two groups were calculated using Student's *t*‐test (two‐tailed) by graphpad prism version 7.00 (San Diego, CA, USA). The statistical significances were at *P* values < 0.05.

## 3 Results

### 3.1 XB1034, XB1034‐NHS, and XB1034‐cetuximab‐TCO

**Fig. 1:**
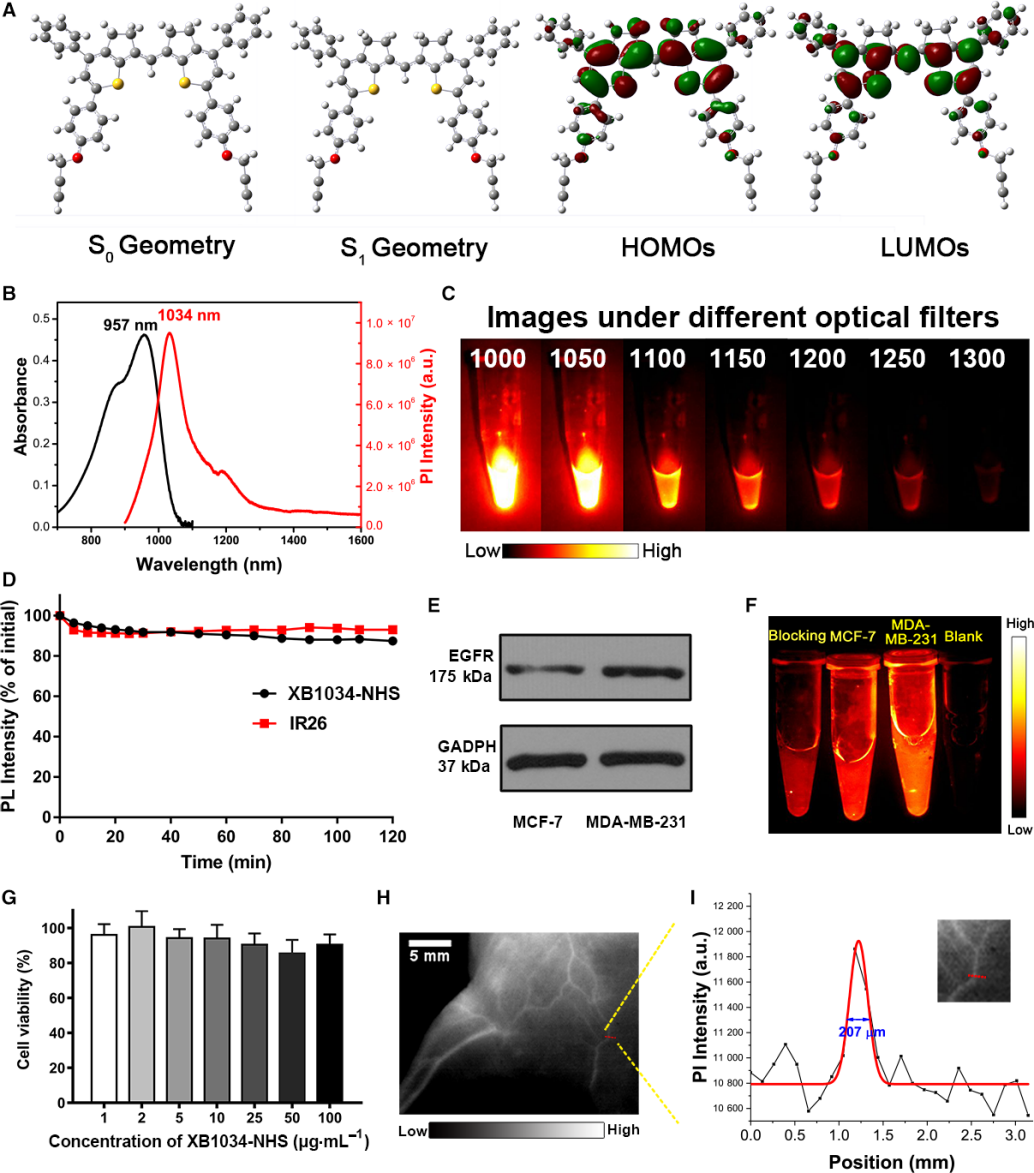
(A) The optimized geometries of *S*_0_ and *S*_1_, HOMOs, and LUMOs were calculated with gaussian 09 software. (B) The absorbance and emission spectra of XB1034 in dichloromethane. (C) Fluorescence signals of XB1034 in sequential long‐pass filters (1000–1300 nm). (D) The photostability of XB1034‐NHS within 2 h. (E, F) The binding ability of XB1034‐cetuximab‐TCO to MDA‐MB‐231 was higher than that to MCF‐7 cells, which is in accordance with the western blot results. (G) The NIH 3T3 cell viability of XB1034‐NHS, error bars indicate ± SD. (H) The vascular imaging of XB1034 in nude mice. (I) The fluorescence intensity profiles (black line) and Gaussian fit across a red line marked in H.

The optimized geometries of *S*_0_ and *S*_1_, HOMOs, and LUMOs of XB1034 are shown in Fig. 1A. The band gap of HOMOs and LUMOs is 1.72 eV, and the calculated emission wavelength was approximately 1000 nm. These calculated properties showed XB1034 an ideal dye for NIR‐II imaging. XB1034 was successfully synthesized and characterized by nuclear magnetic resonance (Figs S1 and S2) and MALDI‐TOF mass spectrometry (Fig. S3). The maximum absorption and emission wavelengths of XB1034 were 957 and 1034 nm in dichloroethane (DCE; Fig. 1B), respectively, which was consistent with gaussian 09 calculations. The molecular absorption coefficient was 3.71 × 10^4^ m ^−1^·cm^−1^ in dichloroethane. The fluorescence QY was 3.2% in DCE with IR26 (QY = 0.5%) as a reference. The fluorescence intensities of XB1034 (10 μm in DCE) were identified with sequential LP filters (1000–1300 nm). The fluorescence signals of XB1034 had been slightly weaker at 1050‐nm long‐pass filter and decreased sharply after 1100 nm (Fig. 1C).

XB1034 was modified with short‐chain polyethylene glycol to achieve water solubility using a high‐performance copper‐catalyzed azide/alkyne cycloaddition reaction, along with a highly reactive NHS ester group. The MALDI‐TOF result of XB1034‐NHS is shown in Fig. S4. After continuous laser excitation for 2 h, the fluorescence intensity of XB1034‐NHS remained > 85% of initial in PBS (Fig. 1D), indicating good photostability *in vitro*. Besides, different concentrations of XB1034‐NHS showed no obvious cytotoxicity in NIH 3T3 fibroblasts (Fig. 1G), implying good biocompatibility. The maximum absorption and emission wavelengths of XB1034‐cetuximab‐TCO are shown in Fig. S6. The fluorescence signals of XB1034‐cetuximab‐TCO in MDA‐MB‐231 cells were higher than MCF‐7 and blocking cells (Fig. 1F), showing the affinity and specificity of XB1034‐cetuximab‐TCO *in vitro*.

### 3.2 NIR‐II fluorescence *in vivo* imaging

**Fig. 2:**
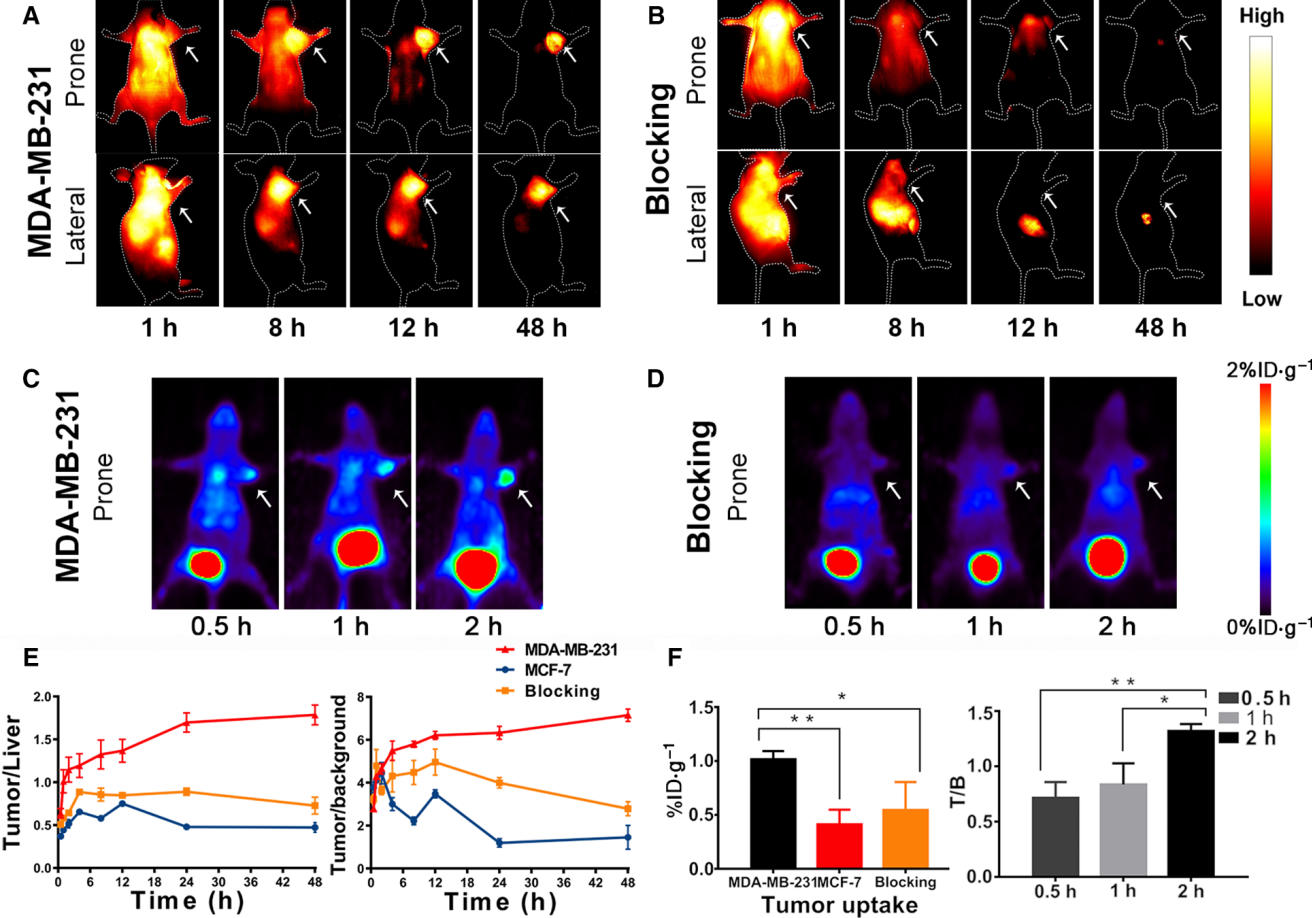
NIR‐II fluorescence imaging taken at 1, 8, 12, and 48 h after an intravenous injection of XB1034‐cetuximab‐TCO of (A) MDA‐MB‐231 and (B) blocked MDA‐MB‐231 (with excessive cetuximab blocking) xenografted models; the PET images of (C) MDA‐MB‐231, and (D) blocked MDA‐MB‐231 tumor‐bearing mice were acquired at 0.5, 1, and 2 h after ^68^Ga‐NETA‐Tz injection. (E) The tumor‐to‐background and tumor‐to‐liver contrasts of the three models at different time points (*n* = 3 per group), which were calculated from the lateral position of the mice. (F) The tumor/blood ratio of MDA‐MB‐231 models at different time points and the tumor uptake of three tumor models at 2 h (*n* = 3 per group). The tumors are indicated by the white arrows. Error bars indicate ± SD. Statistical analysis was performed using Student's *t*‐test (*n* = 3). *P < 0.05 and **P < 0.01.

When the probe (XB1034‐cetuximab‐TCO) was injected into the mice, we immediately acquired images of the blood pool phase (Fig. 1H). The vessel width across the red line is 207 µm, which was calculated from the fluorescence intensity profiles (Fig. 1I), suggesting that NIR‐II imaging shows high spatial resolution for vascular imaging. The MDA‐MB‐231 tumor was clearly seen (Fig. 2A) with rising tumor‐to‐liver (up to 1.79 ± 0.07, *n* = 3; Fig. 2E) and tumor‐to‐background (up to 7.15 ± 0.17) ratios over time. Meanwhile, signals of MCF‐7 tumors (Fig. S10A) were barely detected, and the intensity of MDA‐MB‐231 tumors was reduced sharply in the blocking study (Fig. S10A and Fig. 2B).

### 3.3 PET imaging

After ^68^Ga‐NETA‐Tz injected into the pretargeted MDA‐MB‐231 mice, the tumors were visualized at 0.5 h through the reaction between ^68^Ga‐NETA‐Tz and XB1034‐cetuximab‐TCO (Fig. 2C). Low backgrounds in liver and spleen can also be seen in the pictures at 2 h. The signals in MCF‐7 tumors and blocked tumors were weak (Fig. S10B and Fig. 2D). Using ^68^Ga‐NETA‐cetuximab‐XB1034 to get the direct EGFR‐specific PET images, the radioactivity of blood pool and background signals were constantly high in non‐pretargeted mice (Fig. S11). In the biodistribution studies, ^68^Ga‐NETA‐Tz uptake by kidneys was the highest in all models, indicating that renal excretion is the main clearance pathway (Fig. S12). The concentration of ^68^Ga‐NETA‐Tz in blood was 1.49 ± 0.21% ID·g^−1^ at 0.5 h and rapidly decreased to 0.77 ± 0.05% ID·g^−1^ (*n* = 3 per group) at 2 h postinjection, suggesting a relatively rapid clearance in blood.

### 3.4 NIR‐II image‐guided surgery of tumors

**Fig. 3:**
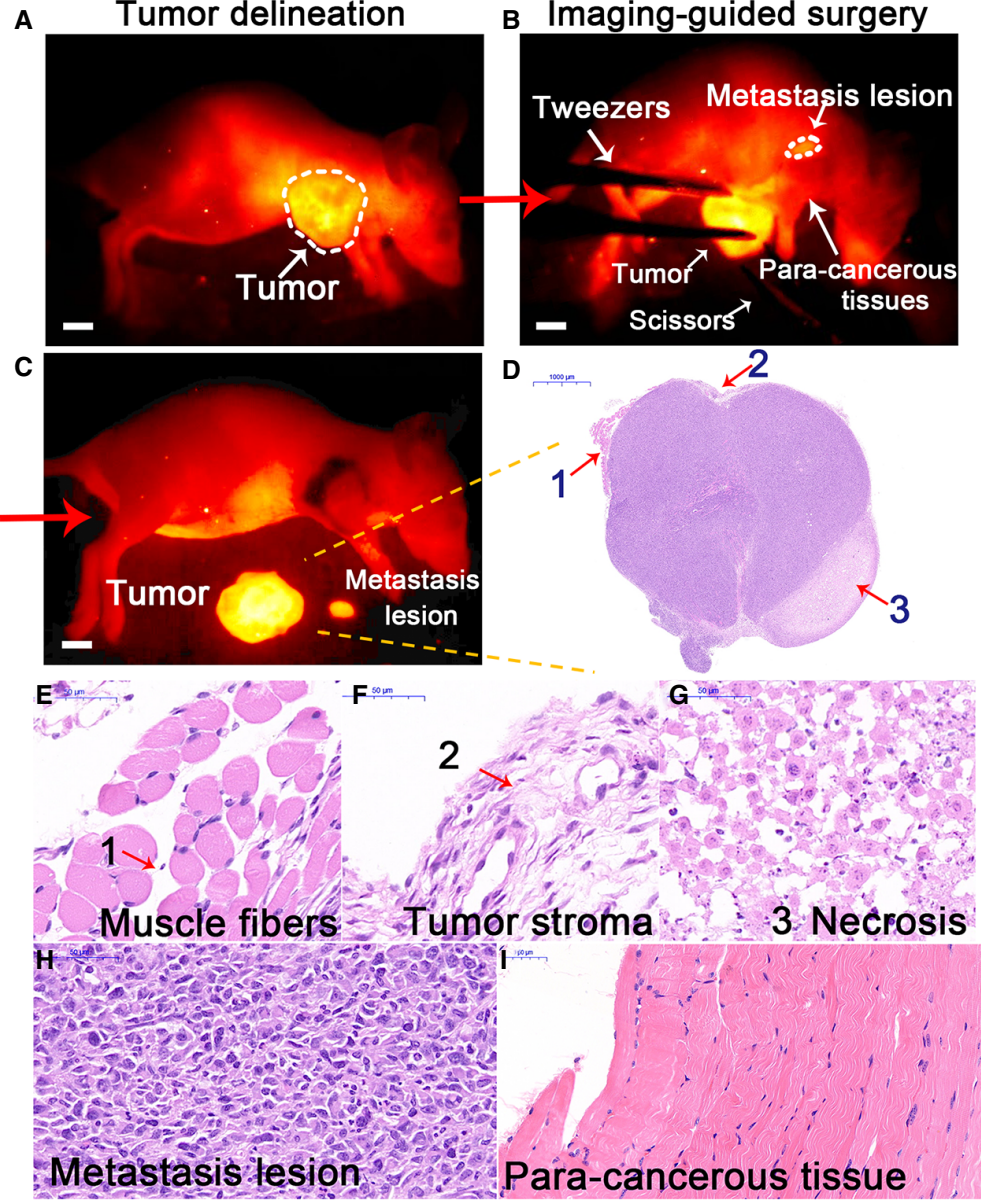
(A–C) With real‐time NIR‐II imaging, MDA‐MB‐231 tumors were delineated and resected thoroughly. (D–G) The histology of the resected tumor was confirmed by H&E staining. (H, I) H&E staining of the metastatic lesions and surrounding tissue. Scale bar (A–C): 5 mm.

After XB1034‐cetuximab‐TCO injection for 60 h (^68^Ga‐NETA‐Tz decayed for 12 h), the delineation of MDA‐MB‐231 tumors was still clear (Fig. 3A). And we performed the resection under dynamic imaging of the mice. During the surgery, the fluorescence enabled the detection and resection of deep and small foci (Fig. 3B,C), which turned out to be metastatic lesions by H&E staining (Fig. 3H). The tumor‐to‐normal ratio reached 6.26 ± 0.40 (*n* = 3), and tumor bed‐to‐normal ratio dropped to 1.74 ± 0.16, which manifests the thorough resection of the tumor using targeted NIR‐II imaging. Histology revealed no malignant cells in the tumor bed (Fig. 3I), indicating an R0 resection.

### 3.5 Sentinel lymph node mapping and imaging‐guided biopsy

**Fig. 4:**
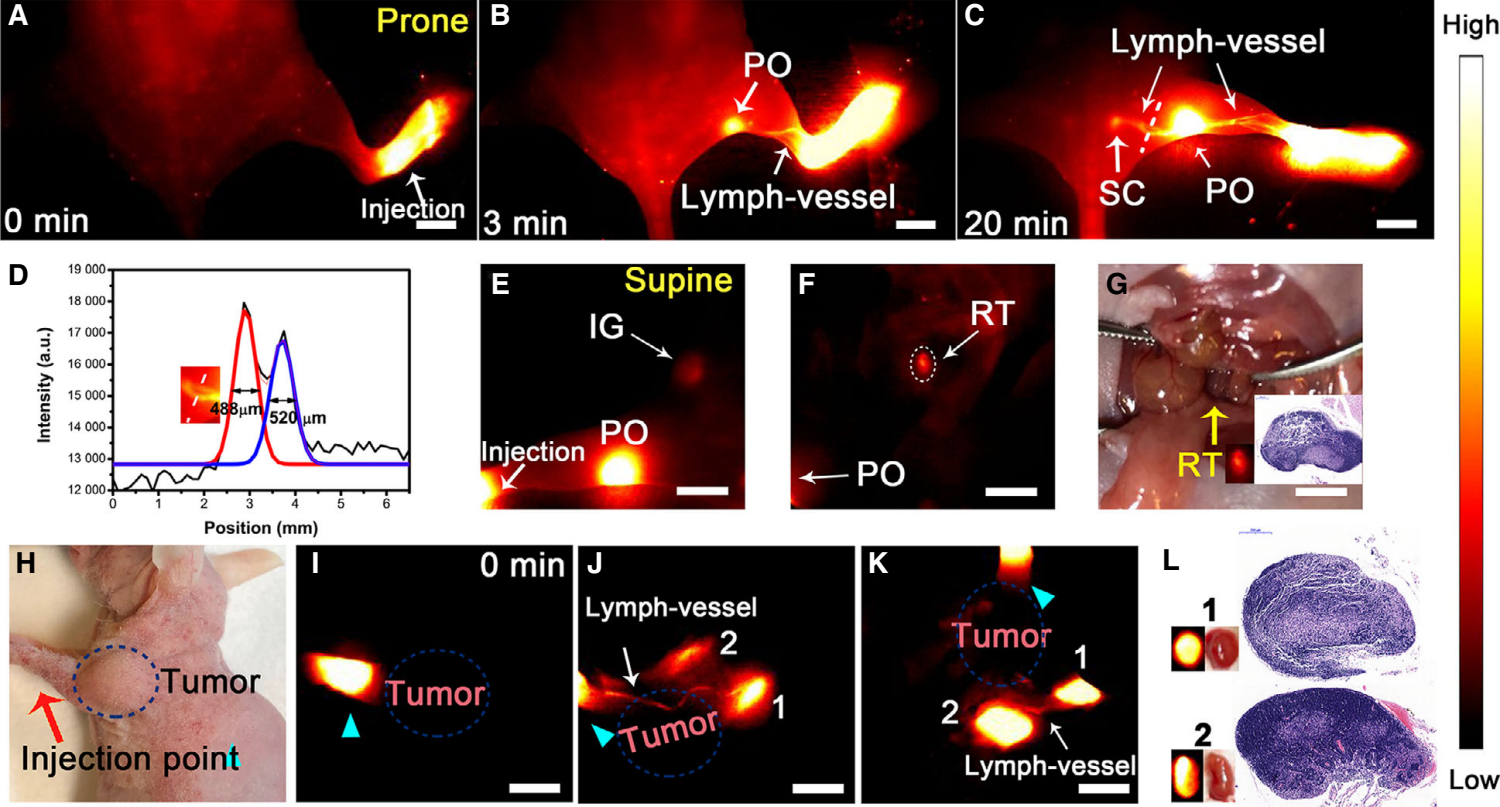
(A–C) Lymph node mapping was performed after subcutaneous injection of the probe into the right foot pad. (D) The fluorescence intensity profiles (black line) and Gaussian function fit (blue and red line) across a white line in C. (E–G) The inguinal and retroperitoneal lymph nodes (IG and RT) could be seen in the supine position. (H–K) Sentinel lymph node mapping of MDA‐MB‐231 tumor‐bearing mice. (L) The biopsy was examined by H&E staining. Red and cyan arrows refered to the injection point. PO, popliteal lymph nodes; SC, sacral lymph nodes. Scale bar: 5 mm.

After XB1034‐cetuximab‐TCO injection into the left hind paw, the lymphatics were visualized in NIR‐II window (Fig. 4A–C). At 3 min postinjection, a popliteal lymph node was identified, and at 20 min postinjection, a sacral lymph node was identified. The diameters of the lymphatics were 488 and 520 µm (Fig. 4D), respectively. In the supine position, inguinal and retroperitoneal lymph nodes were visualized (Fig. 4E,F), which showed improved penetration into deeper tissue in NIR‐II window. Upon injection at the margin of the tumor (Fig. 4H,I), the lymphatics and the axillary lymph nodes became visible (Fig. 4J,K). The histology of the cut tissues was confirmed to be lymph nodes by H&E staining (Fig. 4L).

## 4 Discussion

XB1034 was made water‐soluble with a click reaction. Using XB1034, we constructed a pretargeted PET and NIR‐II imaging probe, XB1034‐cetuximab‐TCO, achieving excellent sensitivity, temporal resolution, and visible tumor delineation during NIR‐II imaging and of PET imaging through a bio‐orthogonal reaction.

In this work, compared with other NIR‐II thiopyrylium salt dyes (Ding *et al.*, 2019), XB1034 had a high molar absorption coefficient, promising quantum yield, low toxicity, and improved stability. Through high‐performance copper‐catalyzed azide/alkyne cycloaddition, XB1034 was bound to NHS ester, the resulting XB1034‐NHS having good water solubility, high fluorescence photostability, and excellent modifiability. This is an alternative to using micelles as nanoparticles (Tao *et al.*, 2013), giving an easy conjugation with a target group to perform high‐efficiency targeted biological imaging (Ding *et al.*, 2019). EGFR‐targeted antibodies were chosen to modify a high specificity to XB1034‐NHS along with TCO. Small vessels were clearly visualized in the blood pool images upon the injection of XB1034‐cetuximab‐TCO, displaying high spatial resolution in NIR‐II window. In EGFR‐targeted tumor imaging, the positive tumors were clearly located with a high SNR (up to 7.15). In contrast to conventional NIR‐I imaging, the NIR‐II probe holds excellent promise for supporting deeper tissue penetration and higher spatial resolution and SNR (Shou *et al.*, 2017; Zhu *et al.*, 2018b).

To overcome the tough issues of antibody‐linked modified probes with high uptake in blood and liver, and improve the clearance of radionuclide in PET imaging, an *in vivo* pretargeting strategy is important (Shi *et al.*, 2018). The biotin/streptavidin system has been used in pretargeting, while it has been damped by the interference of host biotin and biotin enzyme. Recently, the click chemistry between TCO and Tz has displayed the advantages of a rapid reaction, producing a stable product with high selectivity and affinity, and may become a new choice in the application of pretargeting technology in molecular imaging. ^68^Gallium is an ideal and easy‐available positron‐emitting radionuclide for radiopharmaceutical synthesis (Zhang *et al.*, 2018). NETA, a metal chelating agent (Chong *et al.*, 2011; Kang *et al.*, 2012), was selected to coordinate with ^68^Ga for an easy and quick procedure with high labeling yield. Thus, ^68^Ga‐NETA‐Tz was used in pretargeted tumor for PET imaging and the MDA‐MB‐231 tumors were clearly distinguished from other tissues. Because of its small molecule size, ^68^Ga‐NETA‐Tz was quickly cleared by the kidneys and blood. In non‐pretargeted mice, the radioactivity of blood pool was sustained high during the entire imaging. Due to the internalization of some XB1034‐cetuximab‐TCO in 48 h, the uptake value of ^68^Ga‐NETA‐Tz in MDA‐MB‐231 tumors was less than ideal but was much higher than MCF‐7 and blocked tumors at 2 h, identifying the affinity and specificity of pretargeting the probe *in vivo*.

In virtue of the high temporal and spatial resolution, optical imaging is a powerful tool for image‐guided surgery, which allows dynamic feedback for accurate excision of tumors (Sun *et al.*, 2018). Importantly, targeted surgery is especially meaningful for lesions that are hard to differentiate from the surrounding normal tissue. Applying XB1034‐cetuximab‐TCO in NIR‐II image‐guided surgery could provide rapid and accurate feedback to surgeons. During the operation, small lesions and incomplete lesion excision could be identified with NIR‐II real‐time imaging, demonstrating the promise of targeted molecules in NIR‐II fluorescence imaging for precise surgery guidance. The signals dropped sharply after tumor excision, which manifests the thorough resection of the tumor using targeted NIR‐II imaging. SLN mapping has been a gold standard in clinical application in predicting metastasis (Mamounas *et al.*, 2017). Indocyanine green (ICG) is a fluorescence agent, which has been approved by the FDA for 61 years (Hong *et al.*, 2017). Unfortunately, enthusiasm about quantitative lymphatic imaging of ICG is dampened by its poor stability (Rossi *et al.*, 2012). We successfully used XB1034‐cetuximab‐TCO in the normal LN mapping, and the borders of the LNs were figured out clearly and quickly with advantageous temporal and spatial resolution. When using NIR‐II imaging, SLNs were visualized and excised rapidly. And the secondary lymph nodes could be identified and resected by another injection if the histology of SLNs showed positive.

Our study has some drawbacks. The emission wavelength of XB1034 is still limiting for NIR‐II optical imaging due to photon scattering at around 1000 nm. To achieve a higher SNR, redshift of its emission wavelength should be implemented. Moreover, further modification is needed to improve the quantum yield and reduce the imaging dose for better biosafety. This is preliminary research on XB1034, and further study may include the following strategies. First, XB1034 has wide applications, such as vascular imaging, it should be applied in vascular diseases. Second, to further verify the target ability of XB1034‐cetuximab‐TCO in deep metastatic foci, we need to do more investigations with metastatic tumor models, for example, lung and liver metastasis. Because cetuximab is an internalizing antibody, further studies are required to apply noninternalizing antibodies for pretargeting strategy to get better pretargeting PET imaging.

## 5 Conclusion

Overall, we have synthesized an organic NIR‐II dye, XB1034, and constructed a pretargeted PET and NIR‐II imaging methodology. XB1034 manifested promising NIR‐II imaging activity, and the follow‐up injected ^68^Ga probe showed an encourage function on quantification. This targeted dual‐modality probe demonstrated the feasibility of clinical image‐guided operation in real time. We hope to further apply this PET/NIR‐II imaging platform with other large molecular materials, for more widespread and powerful applications in the future.

## Conflict of interest

The authors declare no conflict of interest.

## Author contributions

XZ and BD conceptualized the idea. CQ and HC contributed to methodology. YS and KQ performed formal analysis. XZ and BD underwent investigation. HL, YG, and HF collected resources. XZ wrote the original draft. ZC and XL wrote, reviewed, and edited the manuscript. BD performed visualization. XZ underwent supervision. XL contributed to project administration. XL, XZ, and BD contributed to funding acquisition.

## Supporting information


**Scheme S1.** Synthesis of XB1034, XB1034‐NHS, XB1034‐cetuximab, XB1034‐cetuximab‐TCO and ^68^Ga‐NETA‐cetuximab‐XB1034.
**Fig. S1.** ^1^H NMR spectra of XB1034.

**Fig. S2.** ^13^C NMR spectra of XB1034.

**Fig. S3.** MALDI‐TOF mass spectra of XB1034.

**Fig. S4.** MALDI‐TOF mass spectra of XB1034‐NHS.

**Fig. S5.** The HPLC results of XB1034‐NHS, cetuximab and XB1034‐cetuximab.

**Fig. S6.** Absorption and emission spectra of XB1034‐cetuximab‐TCO.

**Fig. S7.** The radio‐HPLC of ^68^Ga labeling NETA‐Tz.

**Fig. S8.** The ITLC of ^68^Ga‐NETA‐cetuximab‐XB1034.

**Fig. S9.** The immunofluorescence of XB1034‐cetuximab‐CY5 in MDA‐MB‐231 and MCF‐7 cells.

**Fig. S10.** NIR‐II fluorescence and PET imaging of MCF‐7 xenografted models.

**Fig. S11.** The PET imaging of ^68^Ga‐NETA‐cetuximab‐XB1034 in MDA‐MB‐231 mice.

**Fig. S12.** The biodistribution studies of ^68^Ga‐NETA‐Tz.

**Fig. S13.** Popliteal lymph node removal.

**Video S1.** Video of NIR‐II imaged‐guided surgery of tumours.
