# Supplementary figures and images for: A thiopyrylium salt for PET/NIR‐II tumor imaging and image‐guided surgery
Source: Mol Oncol. 2020 Apr 7;14(5):1089–100. doi: 10.1002/1878-0261.12674 (PMC7191196; doi:10.1002/1878-0261.12674)

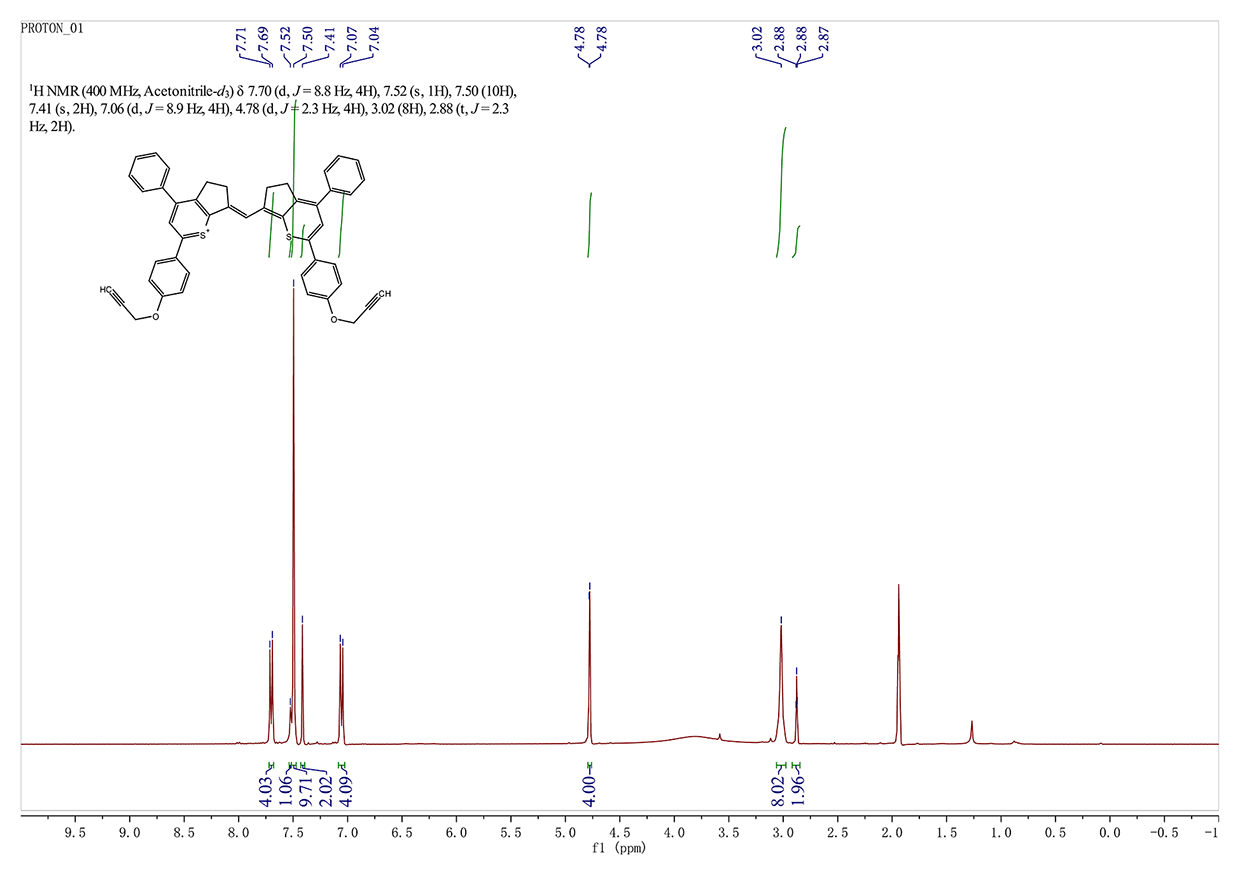

Supplement: Supplementary file 1 — Scheme S1. Synthesis of XB1034, XB1034‐NHS, XB1034‐cetuximab, XB1034‐cetuximab‐TCO and 68Ga‐NETA‐cetuximab‐XB1034. Fig. S1. 1H NMR spectra of XB1034. [file MOL2-14-1089-s001.tif]

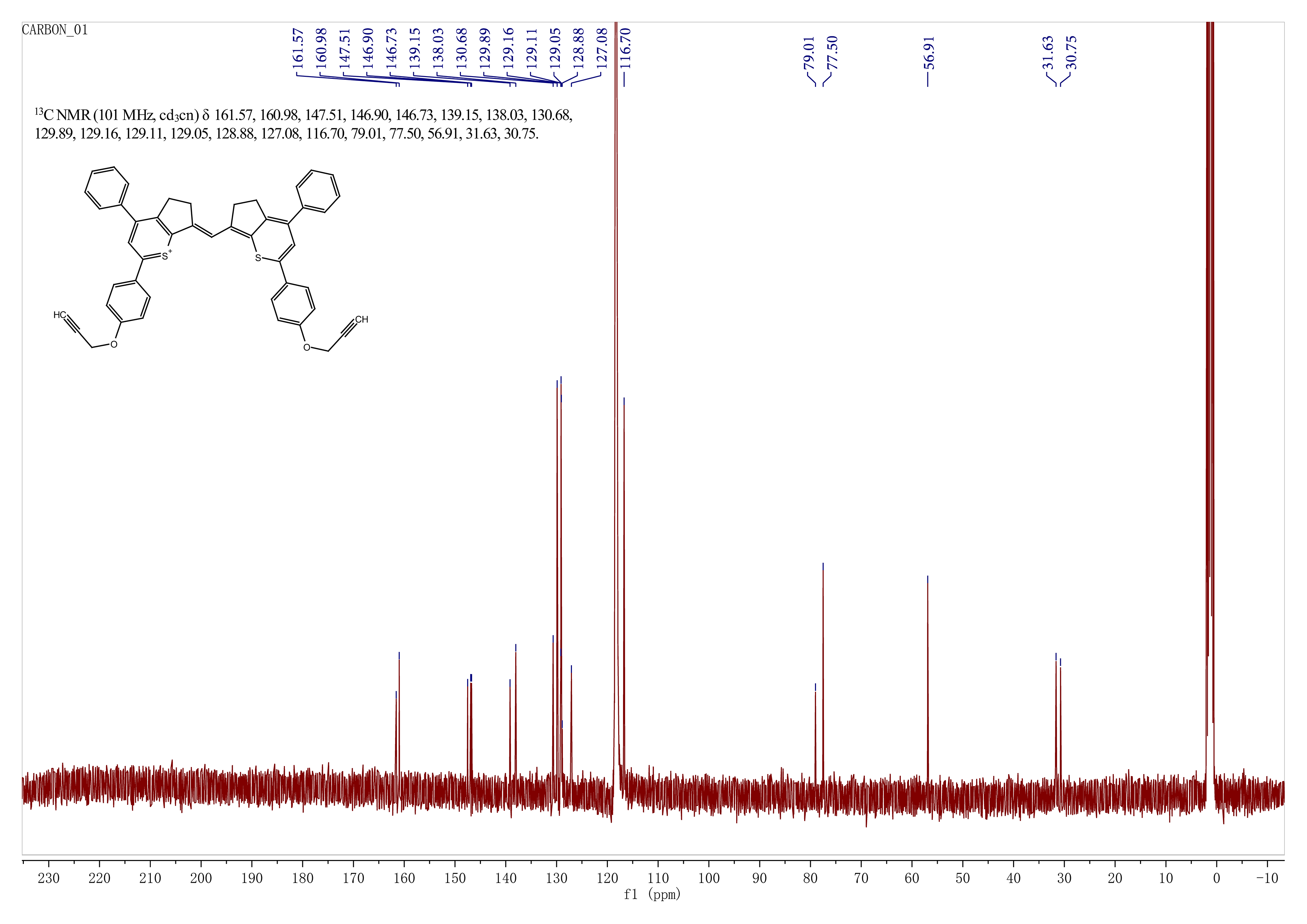

Supplement: Supplementary file 2 — Fig. S2. 13C NMR spectra of XB1034. [file MOL2-14-1089-s002.tiff]

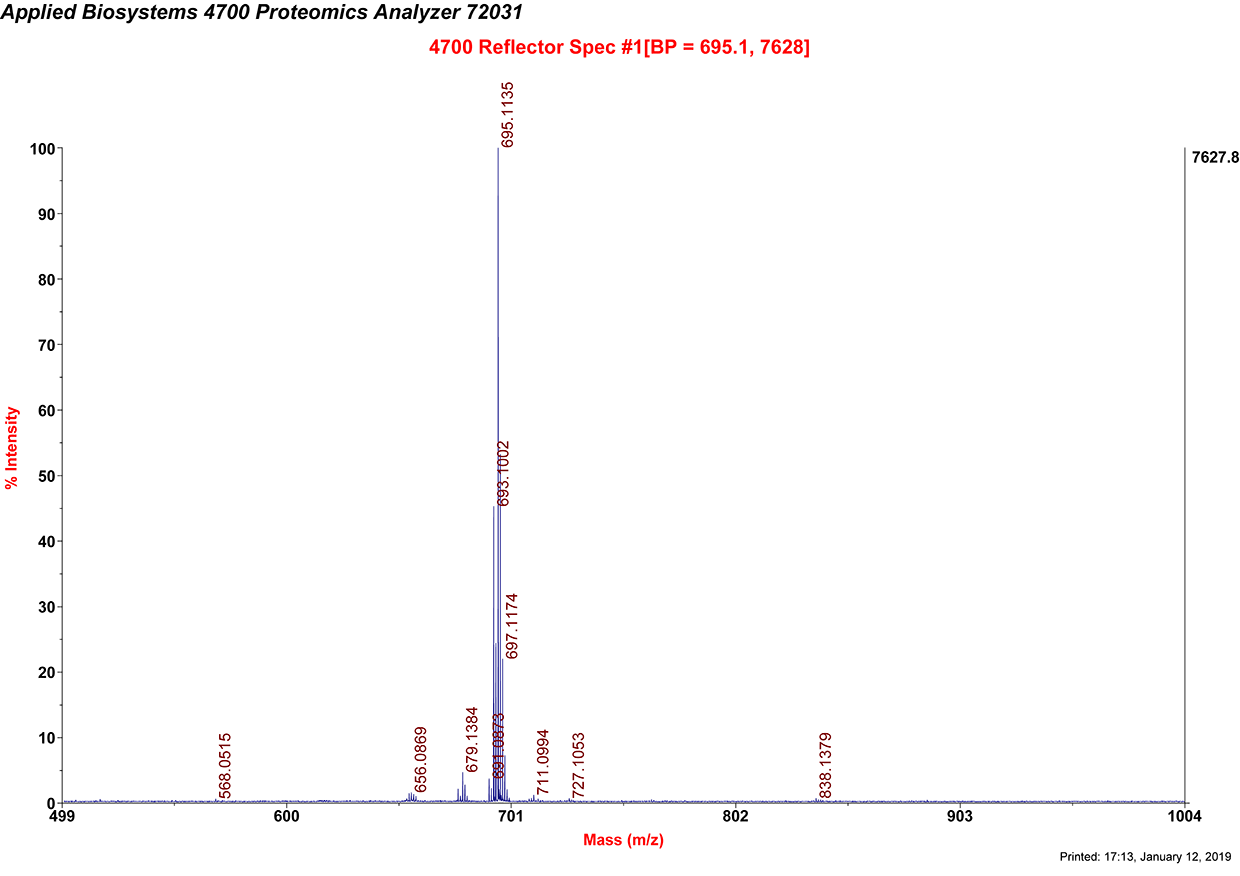

Supplement: Supplementary file 3 — Fig. S3. MALDI‐TOF mass spectra of XB1034. [file MOL2-14-1089-s003.tif]

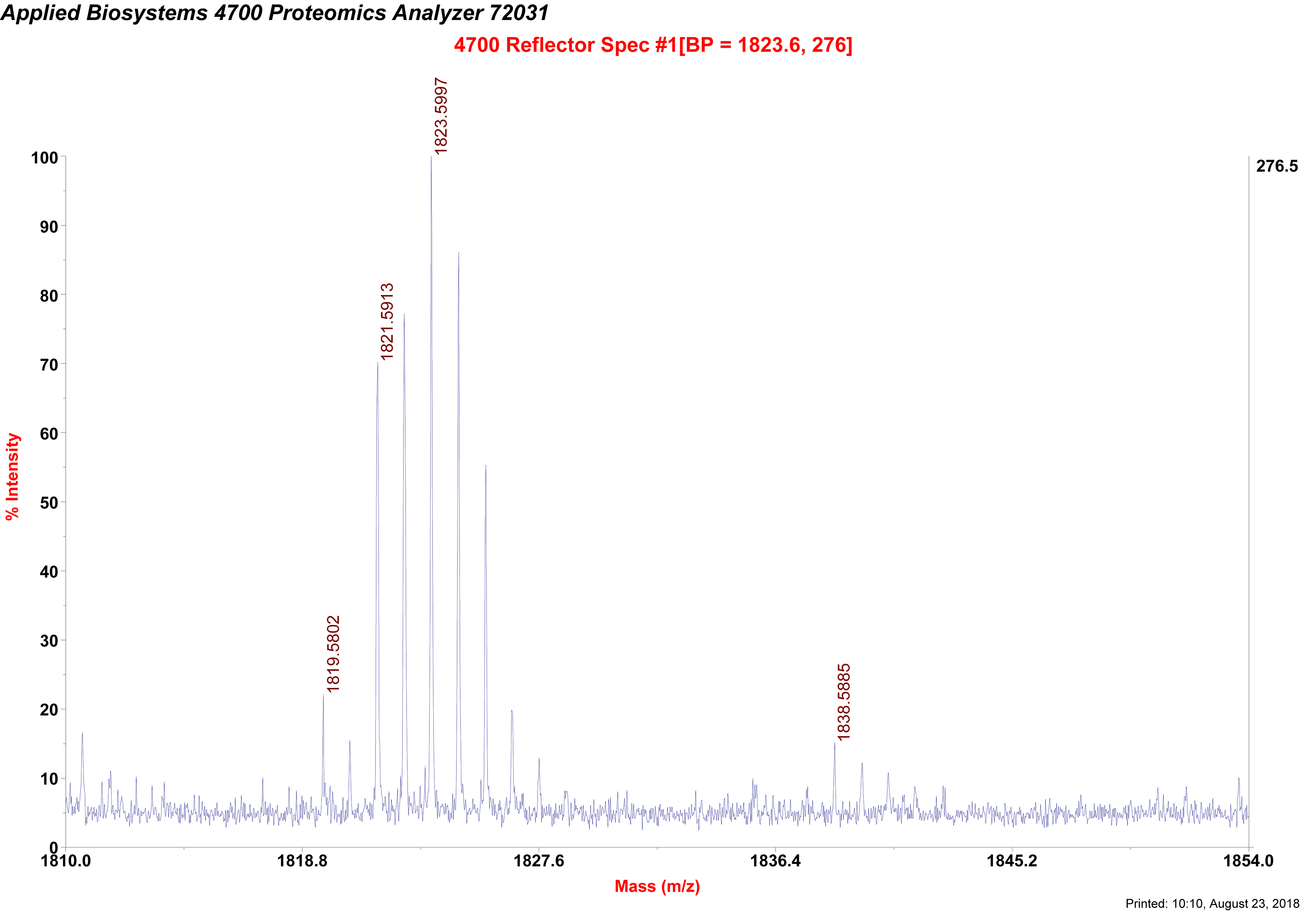

Supplement: Supplementary file 4 — Fig. S4. MALDI‐TOF mass spectra of XB1034‐NHS. [file MOL2-14-1089-s004.tiff]

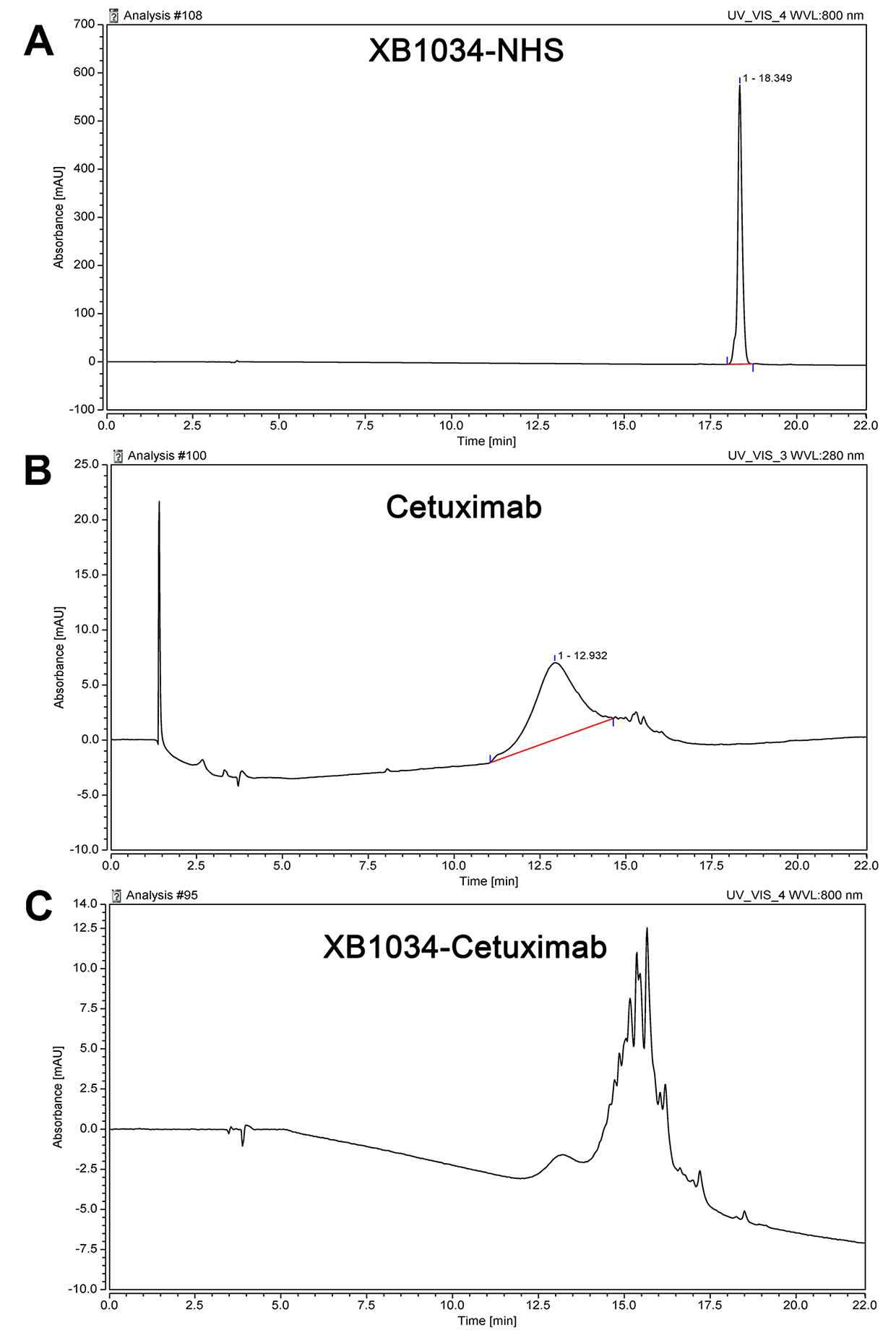

Supplement: Supplementary file 5 — Fig. S5. The HPLC results of XB1034‐NHS, cetuximab and XB1034‐cetuximab. [file MOL2-14-1089-s005.tif]

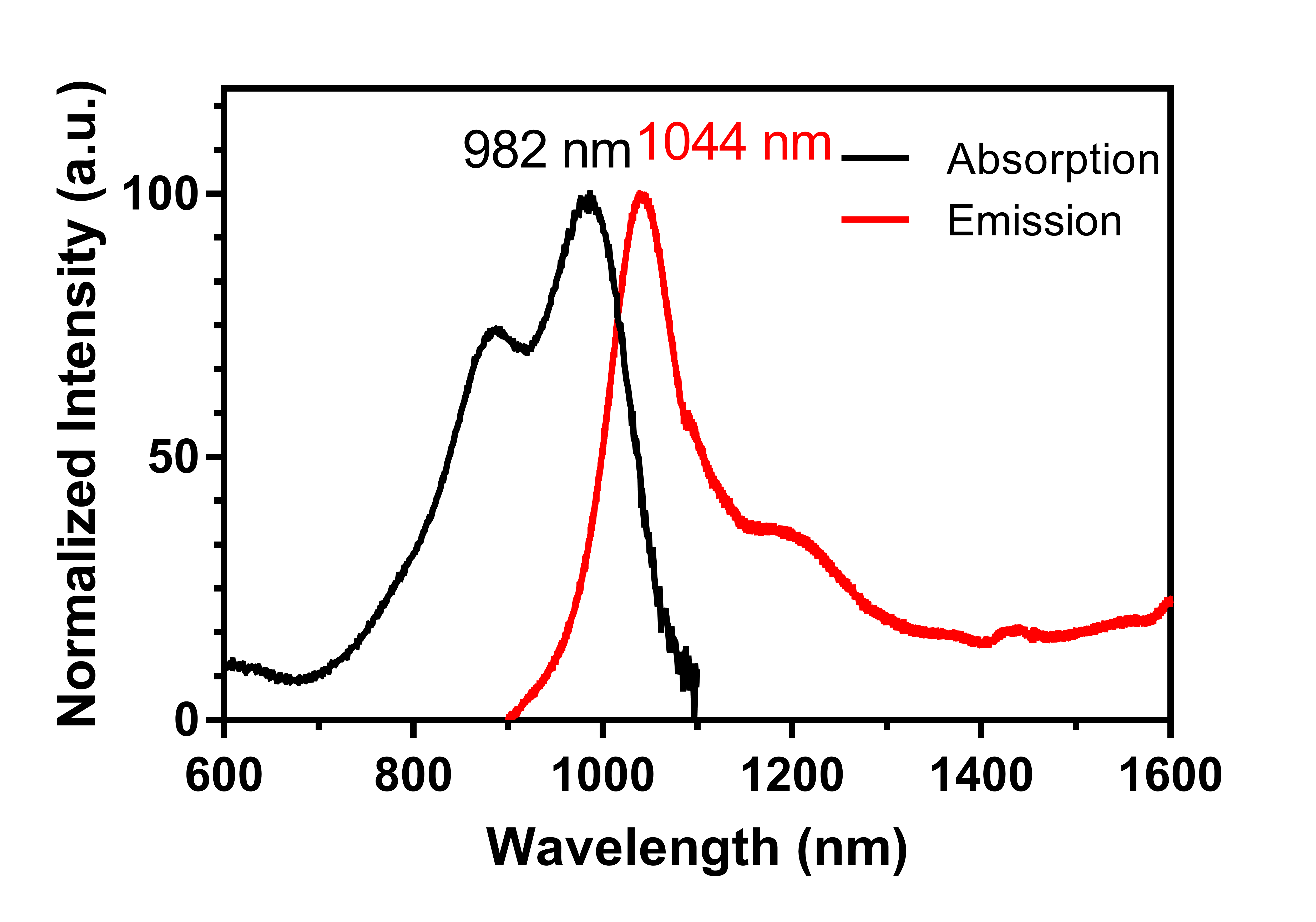

Supplement: Supplementary file 6 — Fig. S6. Absorption and emission spectra of XB1034‐cetuximab‐TCO. [file MOL2-14-1089-s006.tif]

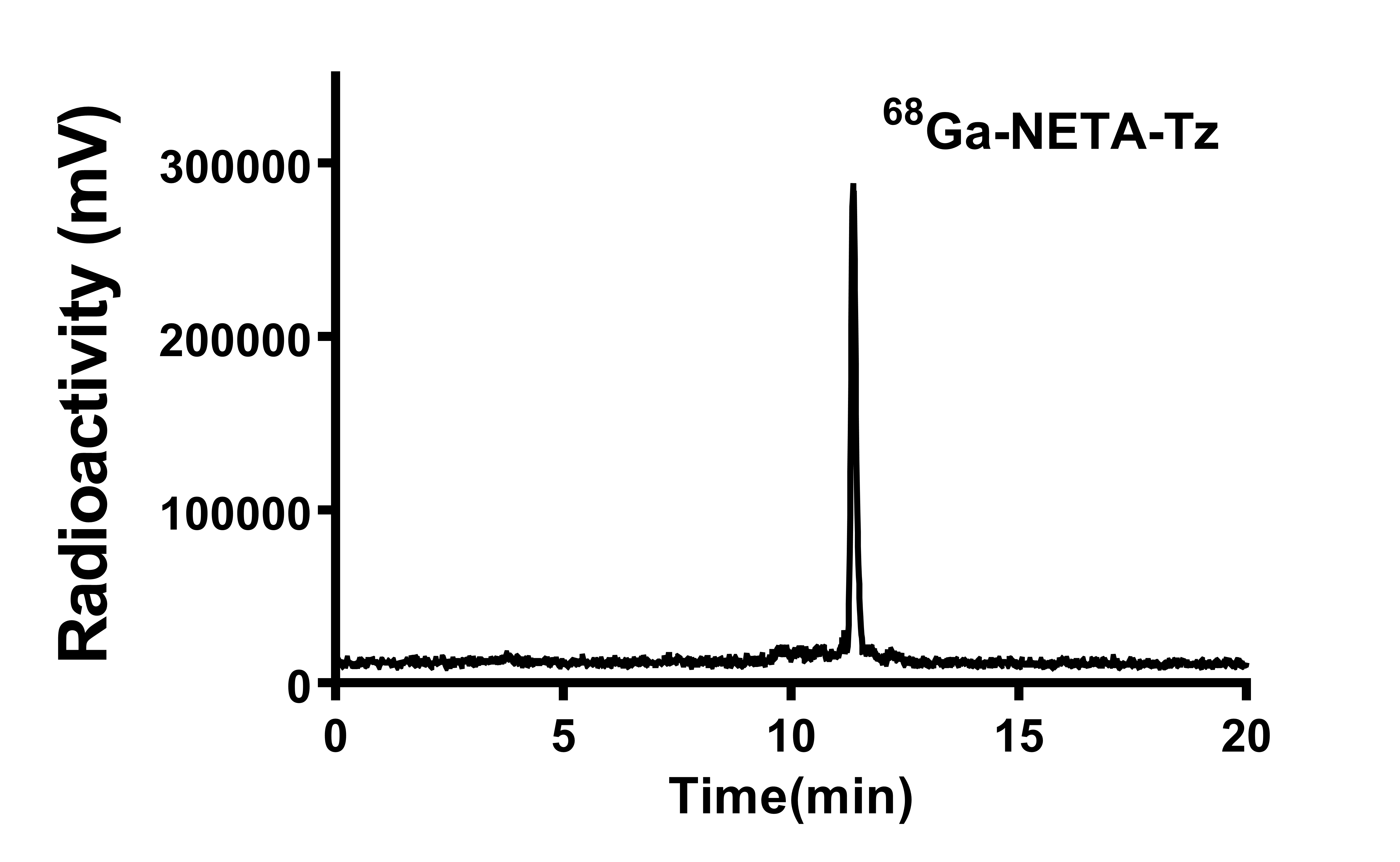

Supplement: Supplementary file 7 — Fig. S7. The radio‐HPLC of 68Ga labeling NETA‐Tz. [file MOL2-14-1089-s007.tif]

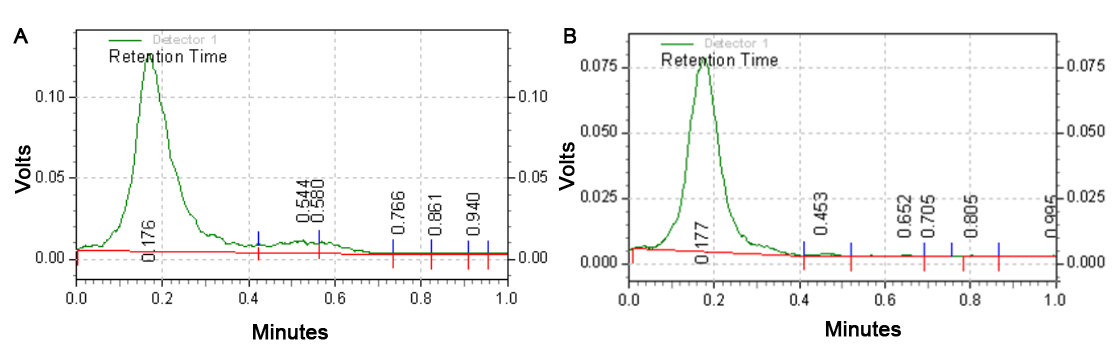

Supplement: Supplementary file 8 — Fig. S8. The ITLC of 68Ga‐NETA‐cetuximab‐XB1034. [file MOL2-14-1089-s008.tif]

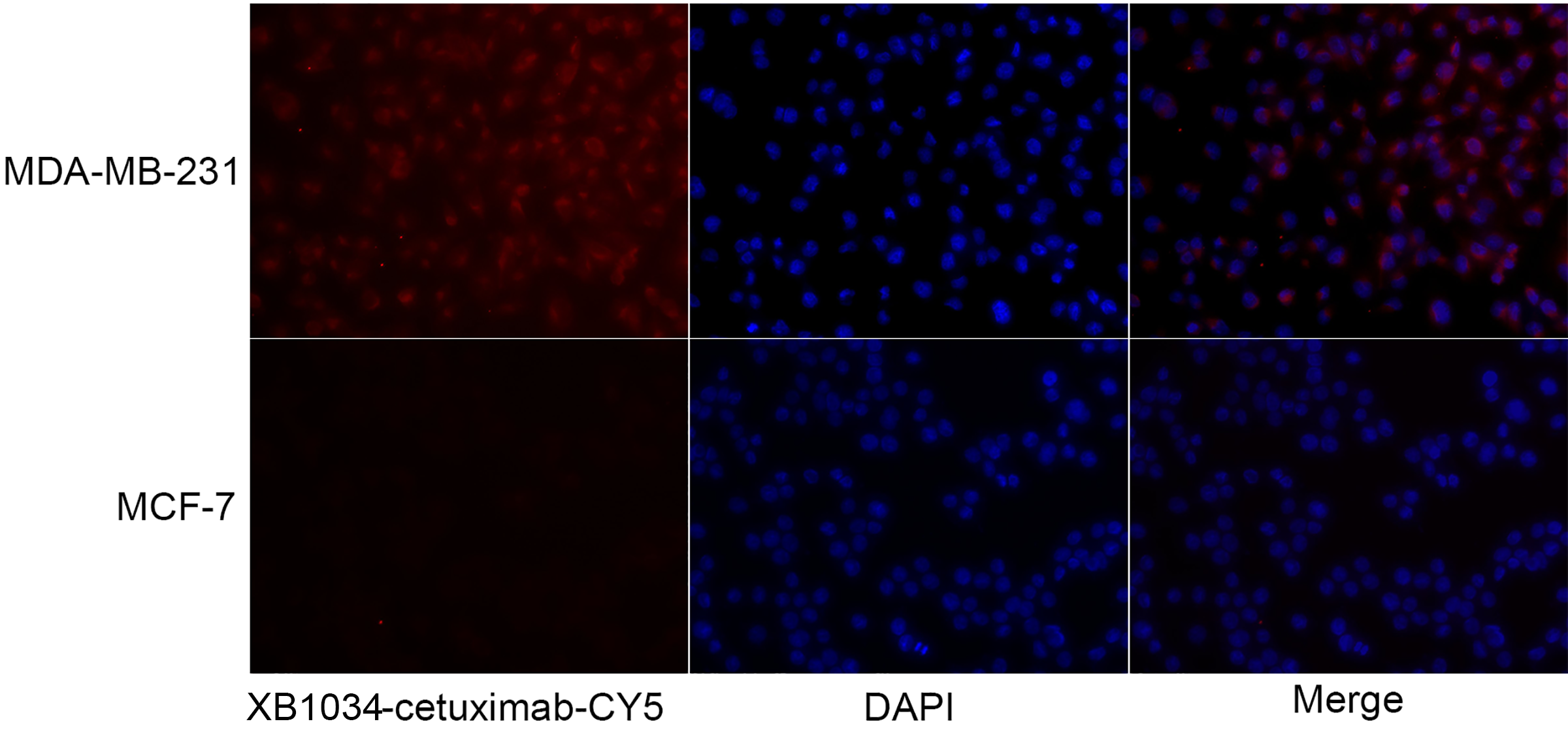

Supplement: Supplementary file 9 — Fig. S9. The immunofluorescence of XB1034‐cetuximab‐CY5 in MDA‐MB‐231 and MCF‐7 cells. [file MOL2-14-1089-s009.tif]

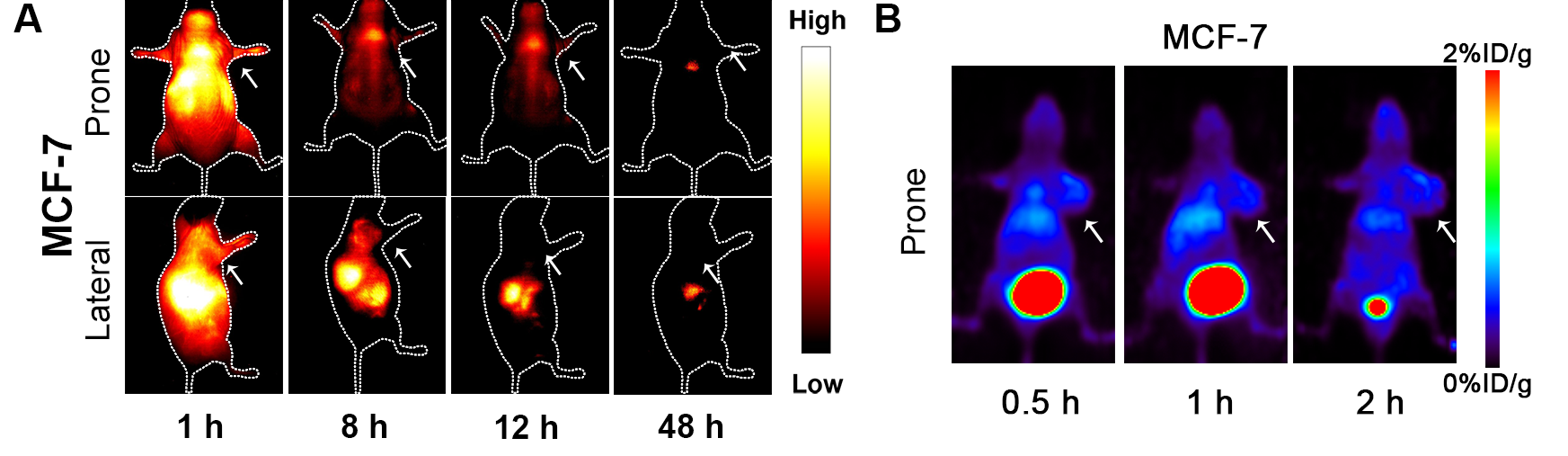

Supplement: Supplementary file 10 — Fig. S10. NIR‐II fluorescence and PET imaging of MCF‐7 xenografted models. [file MOL2-14-1089-s010.tif]

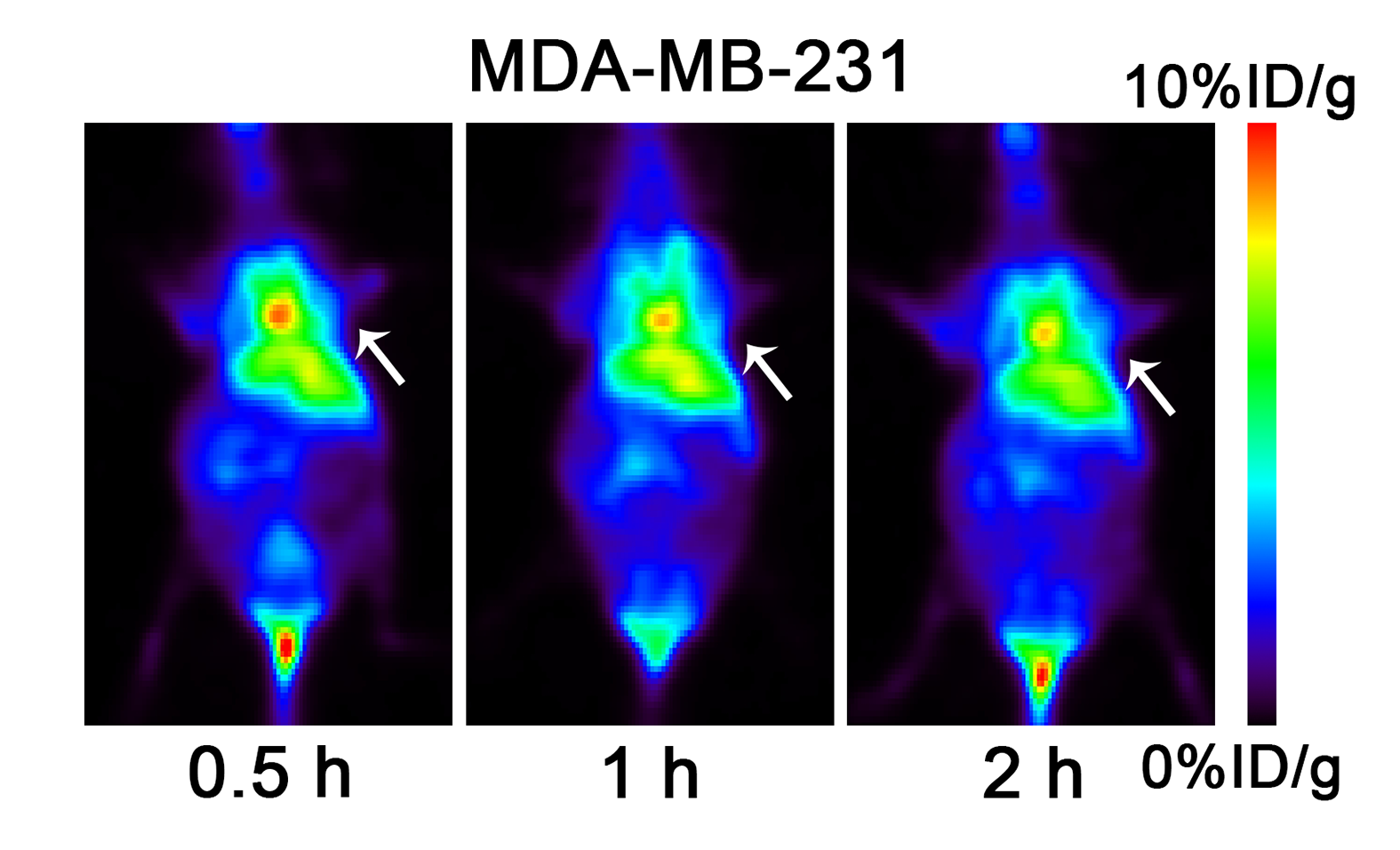

Supplement: Supplementary file 11 — Fig. S11. The PET imaging of 68Ga‐NETA‐cetuximab‐XB1034 in MDA‐MB‐231 mice. [file MOL2-14-1089-s011.tif]

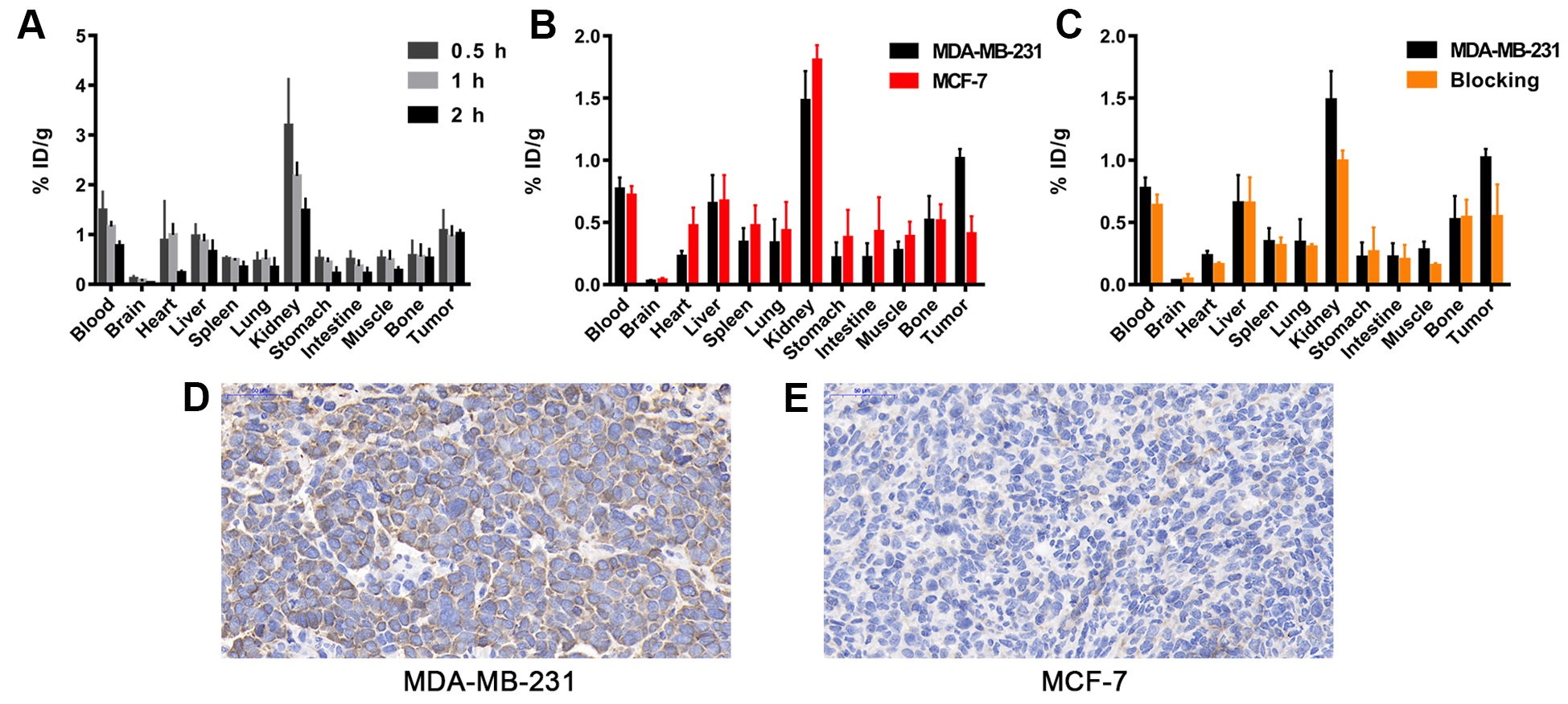

Supplement: Supplementary file 12 — Fig. S12. The biodistribution studies of 68Ga‐NETA‐Tz. [file MOL2-14-1089-s012.tif]

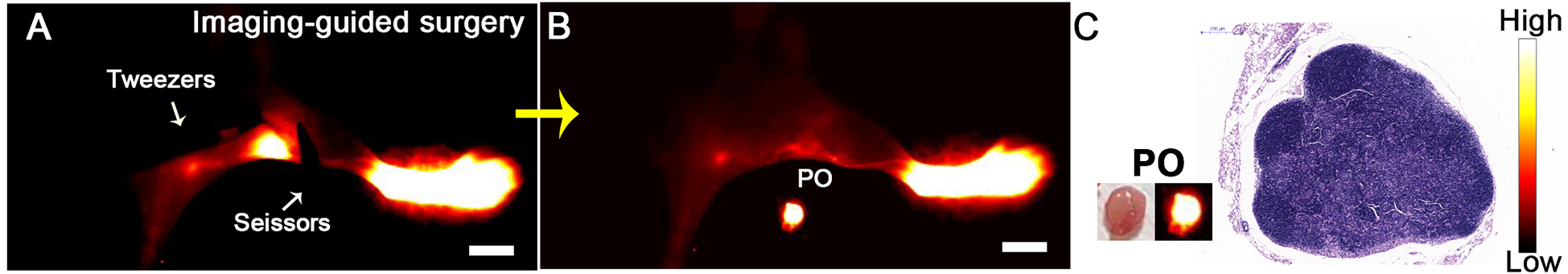

Supplement: Supplementary file 13 — Fig. S13. Popliteal lymph node removal. [file MOL2-14-1089-s013.tif]
