# Supplementary material for: A thiopyrylium salt for PET/NIR‐II tumor imaging and image‐guided surgery
Source: Mol Oncol. 2020 Apr 7;14(5):1089–100. doi: 10.1002/1878-0261.12674 (PMC7191196; doi:10.1002/1878-0261.12674)
Supplement: Supplementary file 15 [file MOL2-14-1089-s015.docx]

Supporting Information

A thiopyrylium salt for PET/NIR-II tumor imaging and image-guided surgery

Xiao Zhang, Bingbing Ding, Chunrong Qu, Huiling Li, Yu Sun, Yongkang Gai, Hao Chen, Hanyi Fang, Kun Qian, Yongxue Zhang, Zhen Cheng*, and Xiaoli Lan*

**1. General methods**

All reagents and solvents were purchased from commercial sources and used without further purification unless indicated otherwise. Flash chromatography was performed on 200–400 mesh silica. Thin layer chromatography (TLC) was performed using Silica gel 60G F_254_ 25 Glass plates and visualized under 254/365 nm ultraviolet light. Nuclear magnetic resonance (NMR) spectra were obtained on Bruker 400 MHz magnetic resonance spectrometer in deuterated solvents and processed in MestReNova software (Mestrelab Research). Matrix-assisted laser desorption/ionization time-of-flight mass spectrometry (MALDI-TOF MS) spectra were obtained on AB SCIEX 5800 TOF/TOF System. High performance liquid chromatography (HPLC) was performed on Dionex Ultimate 3000 HPLC system with in-line Diode Array UV-VIS Detector. UV-VIS absorbance was measured on Agilent Cary 60 UV-Vis Spectrophotometer. The photoluminescence (PL) emission spectrum was obtained on a home-built NIR-II spectroscopy setup. PET imaging was acquired with a small animal PET scanner (BioCaliburn LH^®^, Raycan Technology Co., Ltd., Suzhou, China).

**2. Synthesis scheme**

**Scheme S1.** Synthesis of XB1034, XB1034-NHS, XB1034-cetuximab, XB1034-cetuximab-TCO and ^68^Ga-NETA-cetuximab-XB1034.

**3. The identification of XB1034 and XB1034-NHS**

XB1034 was successfully synthesized and characterized by NMR (Fig. S1 and 2) and MALDI-TOF mass (Fig. S3). The identification of XB1034-NHS was by MALDI-TOF mass (Fig. S4). The molecular weight of XB1034 and XB1034-NHS were 695.2 and 1823.60, respectively, which were in accordance with MALDI-TOF results.


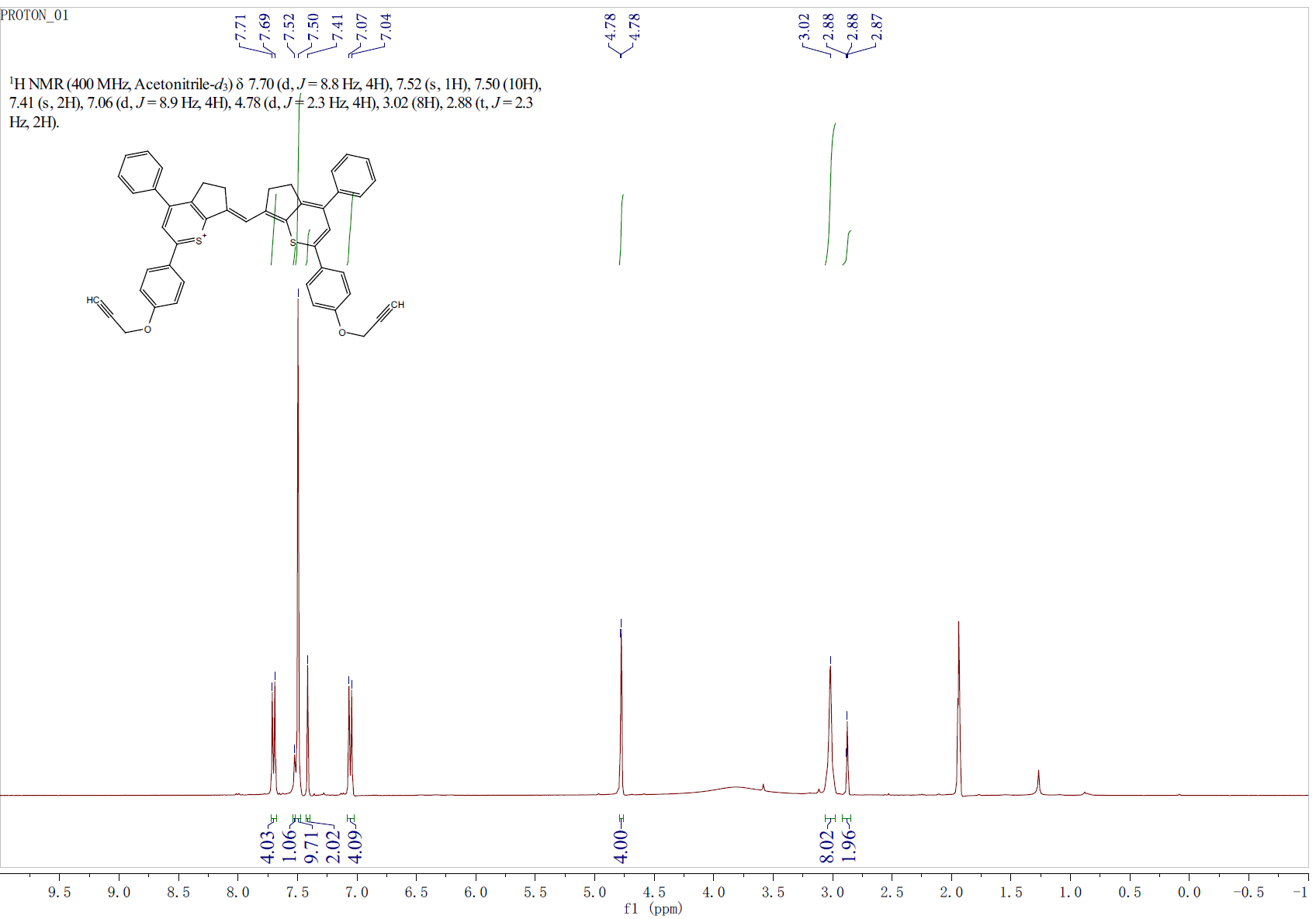


**Fig. S1.** ^1^H NMR spectra of XB1034


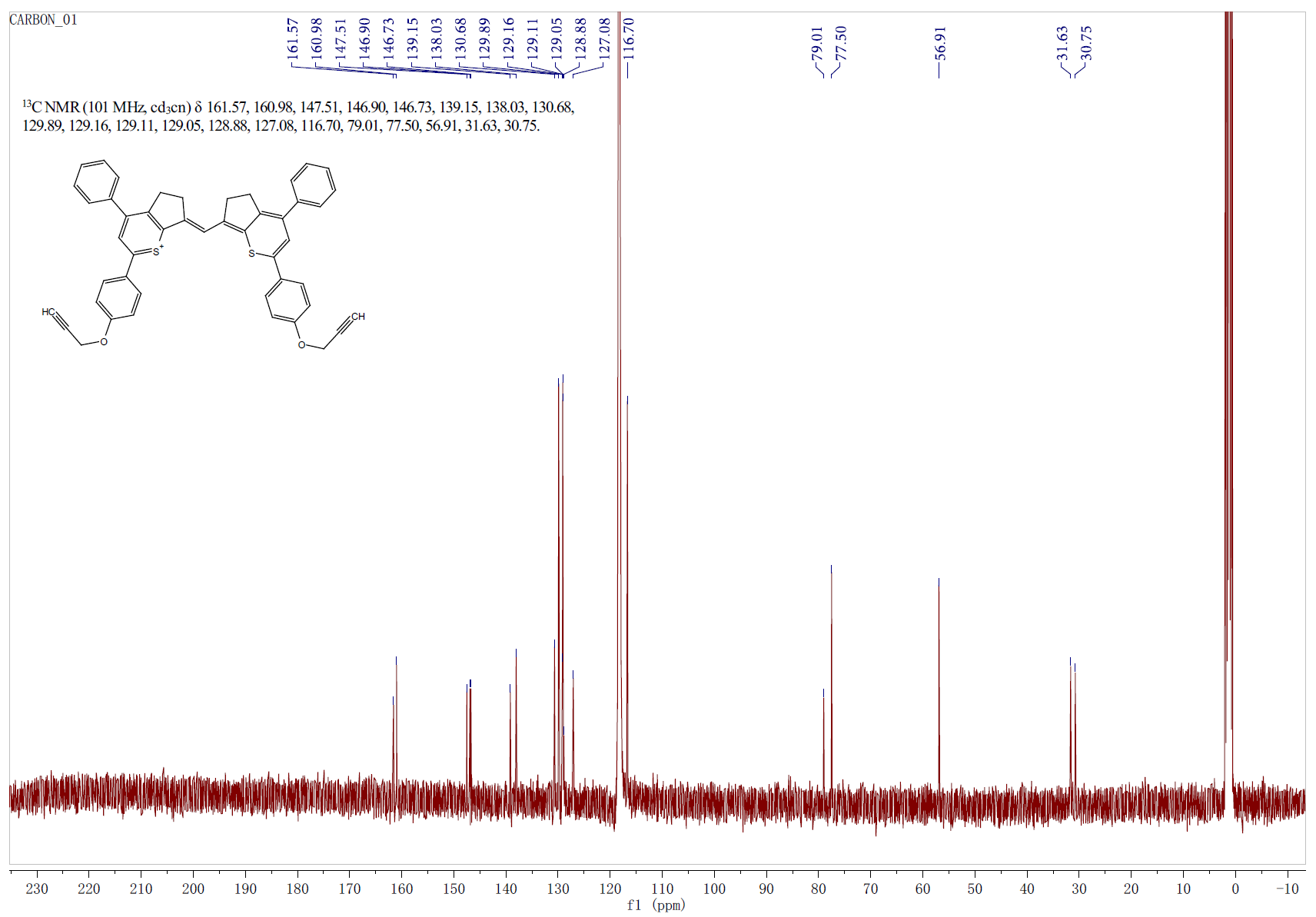
 **Fig. S2**. ^13^C NMR spectra of XB1034


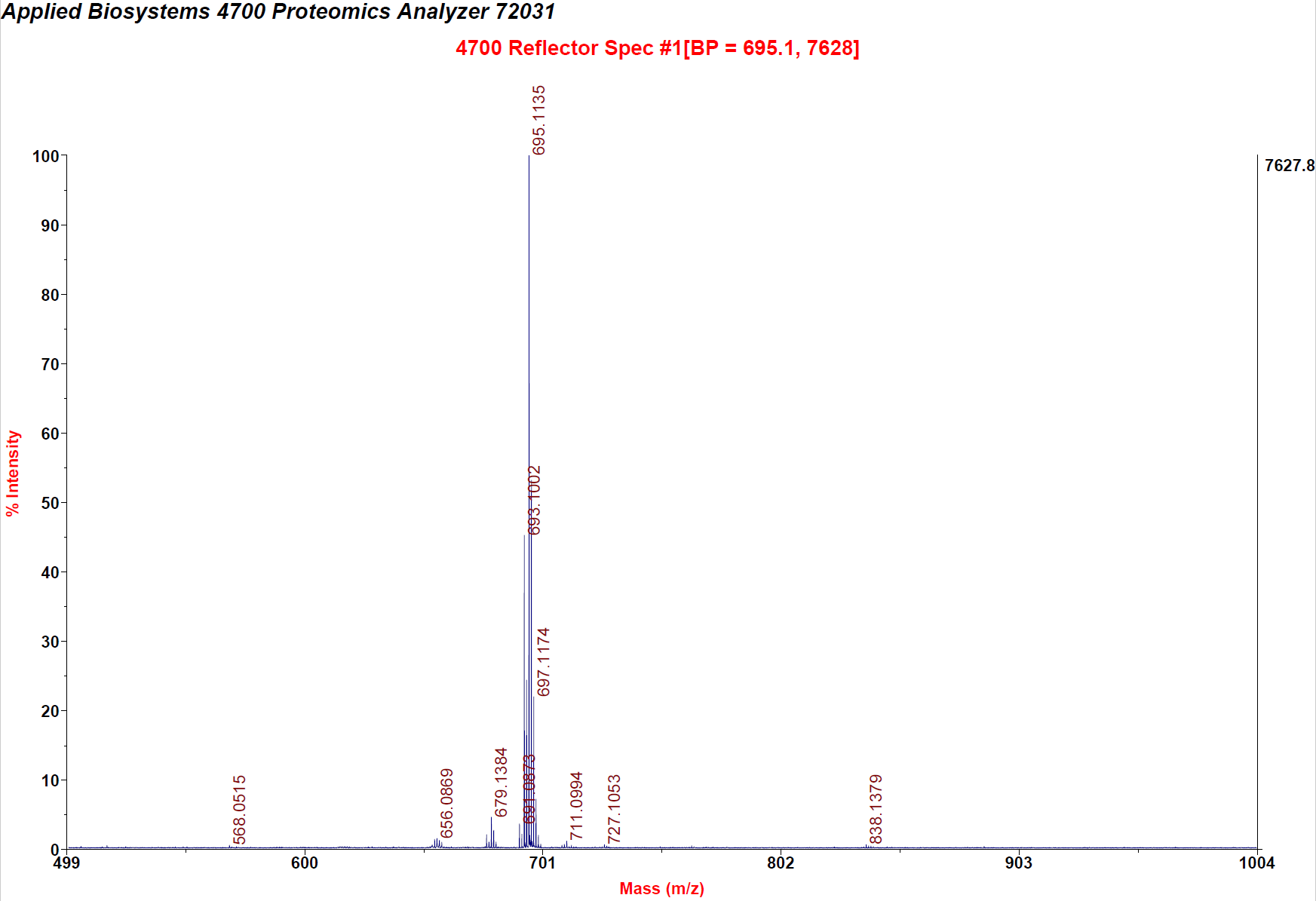


**Fig. S3.** MALDI-TOF mass spectra of XB1034


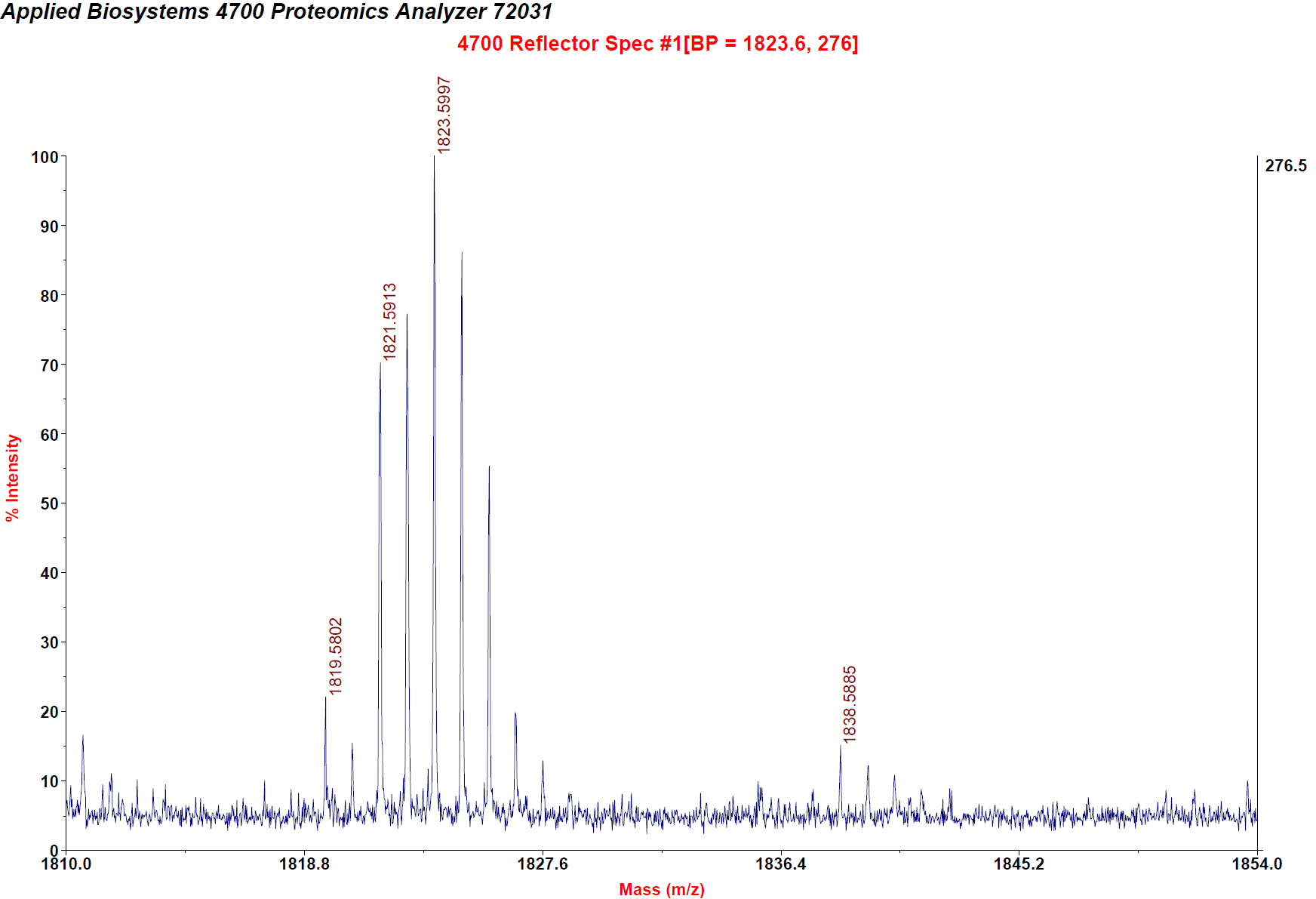


**Fig. S4.** MALDI-TOF mass spectra of XB1034-NHS

**4. The** **method of determining fluorescence quantum yield and the home-built imaging system**

The fluorescence quantum yield (QY) of XB1034 was measured in dichloroethane using **IR-26** (QY = 0.5%) as a reference. Absorbance at 808 nm and fluorescence from 900 to 1600 nm of each dye (5 different concentrations at OD < 0.1) were measured. The integrated fluorescence was plotted against absorbance, and the plots were fitted to a linear function. Comparison of the slopes (K_x_ for testing dye, K_s_ for the reference dye) between each dye and the reference led to the QY. The QY was calculated using the following formula:

$ф_{x}$= $ф_{s} \times\frac{K_{x}}{K_{s}}$

$ф_{x}$: Quantum yield of the testing dye; $ф_{s}$: Quantum yield of **IR-26**

The home-built imaging system is similar to a commercial small-animal imaging system. Simply speaking, a lifting console is placed in a metal box that is protected from light. On the top of the console, a water-cooling NIR-II camera (900-1700 nm, NIRvana 640, Princeton Instrument, USA) and a fiber coupled diode laser (808 nm, 8 W, Lasever Inc., China) are fixed at the top of the box. A 1000 nm long-pass filter is fixed on the near infrared lens before the camera. A collimator is used to distribute laser evenly to a certain area.

**5. The HPLC results of XB1034-NHS, cetuximab, and XB1034-cetuximab**


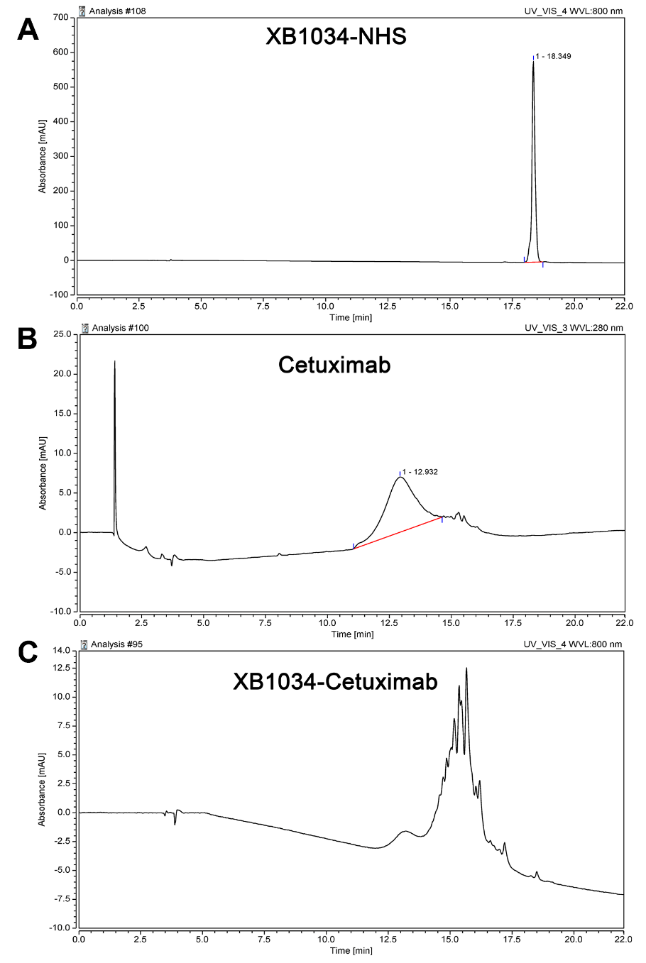


**Fig. S5.** The HPLC results of XB1034-NHS (A), cetuximab (B) and XB1034-cetuximab (C). The retention time of XB1034-cetuximab was advanced from 18.35 to 12.93 min compared with XB1034-NHS.

**6. The maximum absorption and emission wavelengths of XB1034-cetuximab-TCO**

The maximum absorption and emission wavelengths (Fig. S6) of XB1034-cetuximab-TCO were 982 and 1044 nm, respectively. The QY of the XB1034-cetuximab in deionized water was ~0.2%.


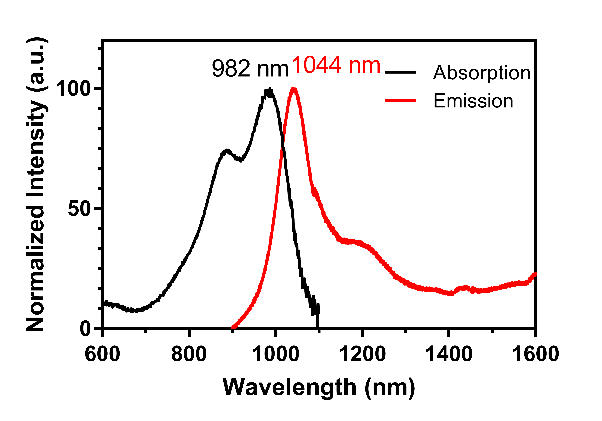


**Fig. S6.** Absorption and emission spectra of XB1034-cetuximab-TCO.

**7. Radiolabeled Probe Preparation and Identification.**

We used 0.05 m HCl to elute ^68^Ga (6 mCi, 500 µL) and sodium acetate buffer (1.25 m, pH = 8.6) was used to adjust the pH to 4.0. NETA-Tz (6 nmol) was added to the reaction and stirred at 90 °C for 10 min to prepare ^68^Ga-NETA-Tz. The radiochemical yield and radiochemical purity of the probe were measured by radio-HPLC using water/acetonitrile as eluent (Fig. S7). The radiochemical yield of ^68^Ga-NETA-Tz was 97.01 ± 2.01% with the specific activity of 35.89 ± 2.15 MBq/nmol and radiochemical purity of 97.11 ± 2.08% (*n* = 3).


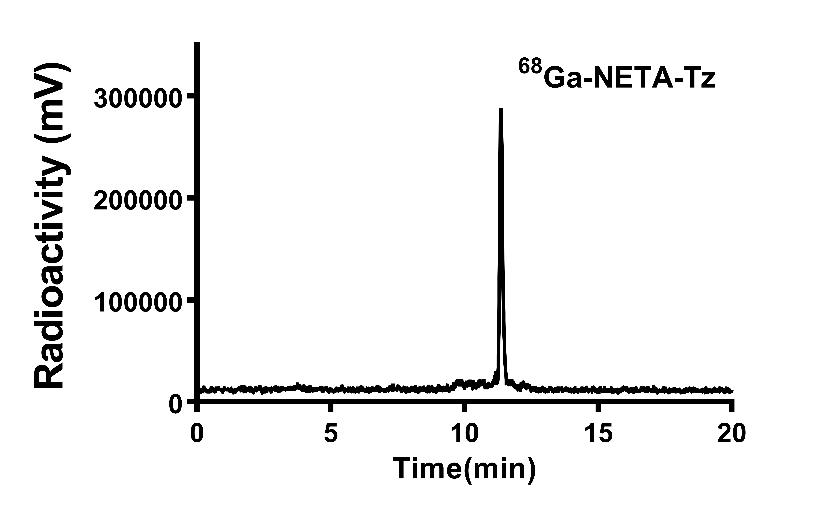


**Fig. S7**. The radio-HPLC of ^68^Ga labeling NETA-Tz.

We used 0.05 m HCl to elute ^68^Ga (111 MBq, 500 µL) and sodium acetate buffer (1.25 m, pH = 8.6, 150 µL) was used to adjust the pH to 4.0. NETA-Tz (3 nmol) was added to the reaction and stirred at 90 °C for 10 min to prepare ^68^Ga-NETA-Tz. After cooling to room temperature, XB1034-cetuximab-TCO (65 µg) was added to ^68^Ga-NETA-Tz, and vibrated for 20 min to prepare ^68^Ga-NETA-cetuximab-XB1034. The product was purified using a PD-10 gel column (General Electric, Fairfield CT, USA). The radiochemical yield and radiochemical purity of the probe were measured by thin layer chromatography (ITLC) using 0.01 m PBS as the developing solvent system.

The radiochemical yield of ^68^Ga-NETA-cetuximab-XB1034 was 90.01 ± 2.23% (Fig. S8A) with a specific activity of 6.94 ± 0.59 MBq/nmol. After purification (Fig. S8B), the radiochemical purity of ^68^Ga-NETA-cetuximab-XB1034 reached 96.02 ± 1.64% (*n* = 3).

***
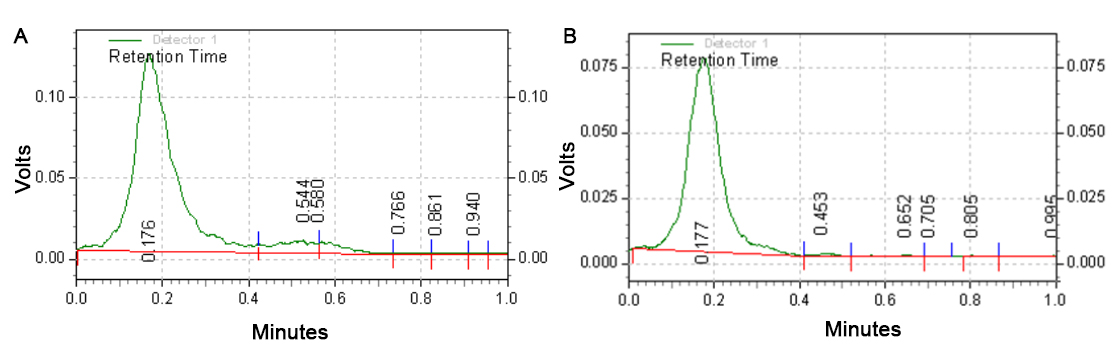
***

**Fig. S8.** The ITLC of ^68^Ga-NETA-cetuximab-XB1034 before (A) and after purification (B)

**8.** **Binding of XB1034-cetuximab-CY5 to EGFR *in vitro***

XB1034-cetuximab was reacted with CY5-NHS to form XB1034-cetuximab-CY5. After blocked in 1% bovine serum albumin (BSA) for 1 h, MDA-MB-231 and MCF-7 cells were incubated with XB1034-cetuximab-CY5 (4.5 μg/ml) overnight, respectively, followed by staining with 4-6-diamidino-2-phenylindole (DAPI) for 4 min. At last, the samples were observed under a confocal microscope. This validated the binding of XB1034-cetuximab to EGFR-positive cells indirectly.


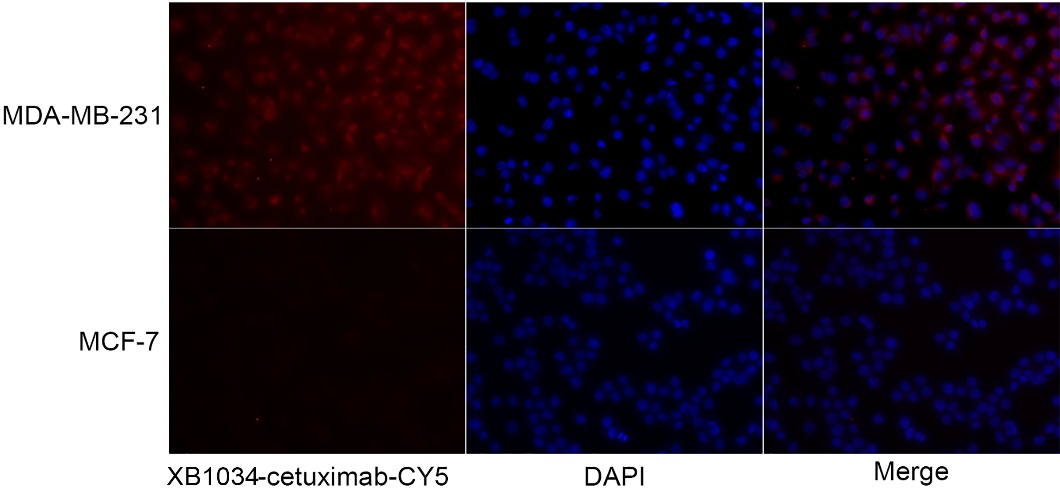


**Figure S9.** The immunofluorescence of XB1034-cetuximab-CY5 in MDA-MB-231 and MCF-7 cells.

**9. NIR-II fluorescence and PET imaging of MCF-7 xenografted models**


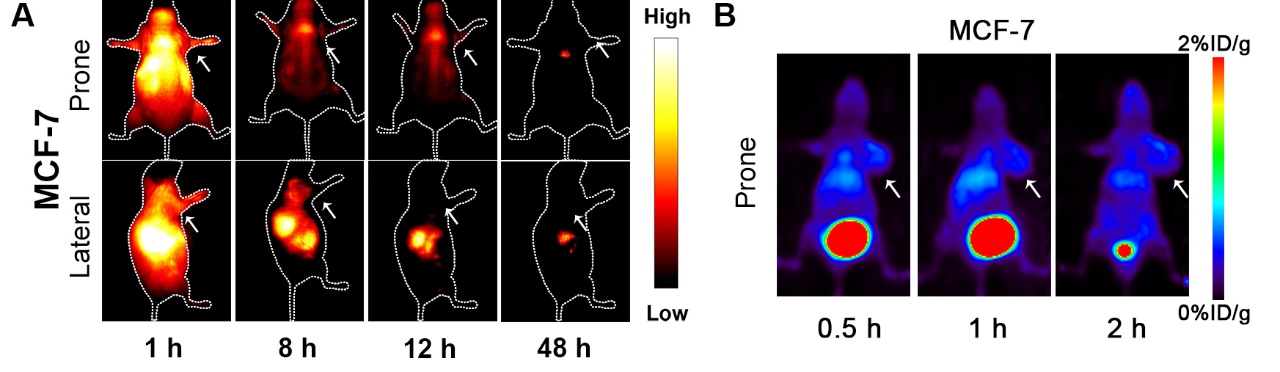


**Fig. S10.** NIR-II fluorescence imaging (A) and PET imaging (B) of MCF-7 xenografted models. The tumors are indicated by the white arrows.

**10. The PET imaging of ^68^Ga-NETA-cetuximab-XB1034 in MDA-MB-231 mice**

As shown in Fig. S11, high blood-pool activity and background uptake were evident constantly in the mice. The images of the livers were visualized clearly, which confirmed that the *in vivo* clearance of the probe is mainly through the hepatic routes. Besides, the probe uptakes in MDA-MB-231 tumors were indistinguishable from the background even at 2 h.

**
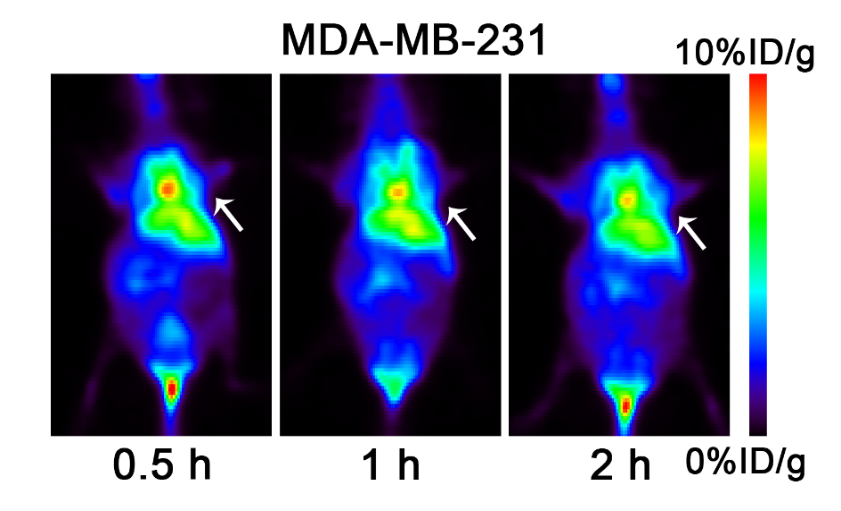
**

**Fig. S11.** The PET imaging of ^68^Ga-NETA-cetuximab-XB1034 in MDA-MB-231 mice. The tumors are indicated by the white arrows.

**11.** **The biodistribution studies of ^68^Ga-NETA-Tz in MDA-MB-231 and MCF-7 bearing mice**

In the biodistribution studies, the uptake of ^68^Ga-NETA-Tz by the kidneys was the highest in all models, indicating that renal excretion is the main clearance pathway (Fig. S12). The concentration of ^68^Ga-NETA-Tz in blood was 1.49 ± 0.21% ID/g at 0.5 h and rapidly decreased to 0.77 ± 0.05% ID/g (*n* = 3 per group) at 2 h post-injection, suggesting a relatively rapid clearance in blood. The IHC staining of MDA-MB-231 and MCF-7 tumors are shown in Fig. S12D and E, respectively.


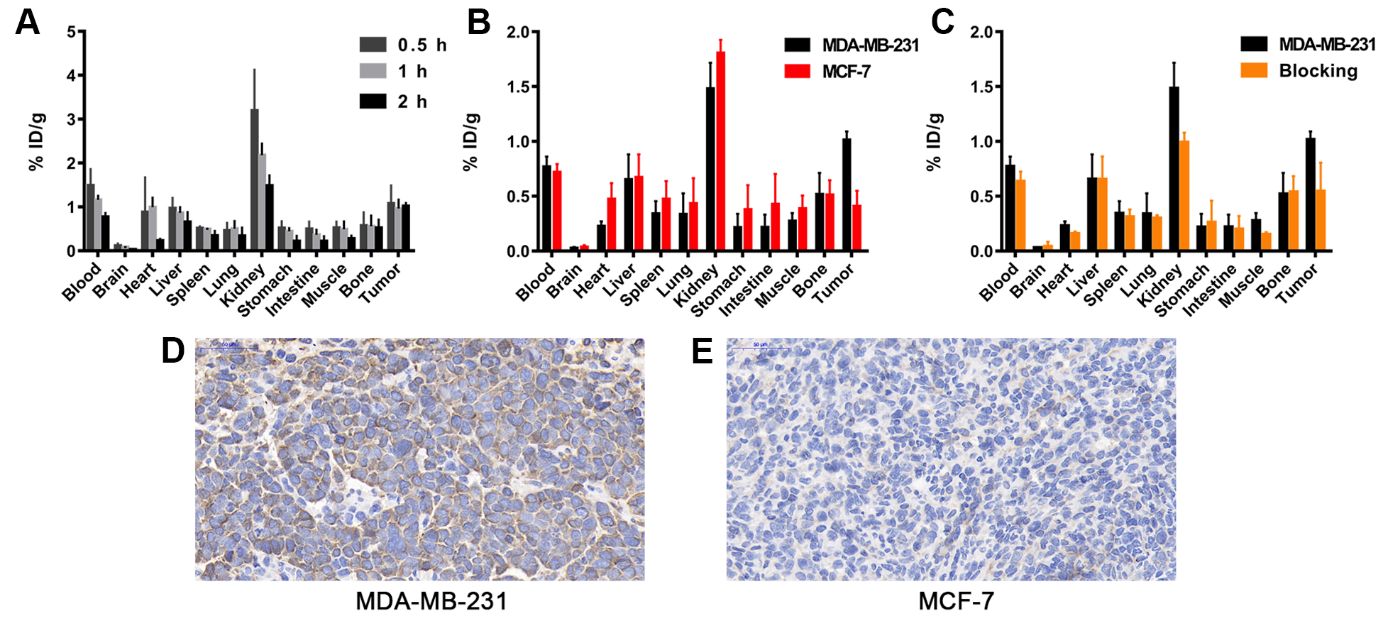


**Fig. S12**. After XB1034-cetuximab-TCO injected for 48 h, ^68^Ga-NETA-Tz were injected to access the organic uptake in MDA-MB-231 mice at different time points (A, *n* = 3 per group). The contrast of MDA-MB-231 and MCF-7 mice at 2 h (B, *n* = 3 per group). The blocking study of MDA-MB-231 tumor bearing mice at 2 h (C, *n* = 3 per group). The tumors were resected to verify the expression of EGFR by immunohistochemistry (IHC, D and E).

**12.** **Popliteal lymph node removal**

We also removed the popliteal lymph node under NIR-II imaging. The lymph node/background ratio (SBR) was 12.82 ± 0.76 (*n* = 3 per group) before the removal (Fig. S13A). After sentinel lymph node removed, the origin lymph node/background ratio dropped to 2.39 ± 0.26 (*n* = 3 per group, Fig. S13B). The histology of the cut tissues was confirmed to be lymph nodes by H&E staining (Fig. S13C).


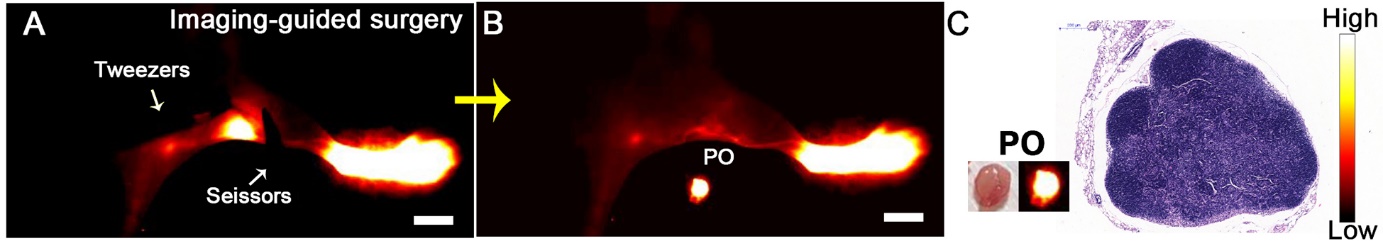


**Fig. S13**. Under NIR-II imaging, the popliteal lymph node was clearly delineated and separated from the body in real time (A, B). The histology of the lymph node was confirmed by H&E staining (C). Scale bar: 5 mm.
